# Impact of nanoparticles on amyloid β-induced Alzheimer’s disease, tuberculosis, leprosy and cancer: a systematic review

**DOI:** 10.1042/BSR20220324

**Published:** 2023-02-07

**Authors:** Ayon Chakraborty, Saswati Soumya Mohapatra, Subhashree Barik, Ipsita Roy, Bhavika Gupta, Ashis Biswas

**Affiliations:** School of Basic Sciences, Indian Institute of Technology Bhubaneswar, Bhubaneswar, India

**Keywords:** Alzheimer's disease, Cancer, Drug delivery, Leprosy, Nanoparticles, Tuberculosis

## Abstract

Nanotechnology is an interdisciplinary domain of science, technology and engineering that deals with nano-sized materials/particles. Usually, the size of nanoparticles lies between 1 and 100 nm. Due to their small size and large surface area-to-volume ratio, nanoparticles exhibit high reactivity, greater stability and adsorption capacity. These important physicochemical properties attract scientific community to utilize them in biomedical field. Various types of nanoparticles (inorganic and organic) have broad applications in medical field ranging from imaging to gene therapy. These are also effective drug carriers. In recent times, nanoparticles are utilized to circumvent different treatment limitations. For example, the ability of nanoparticles to cross the blood−brain barrier and having a certain degree of specificity towards amyloid deposits makes themselves important candidates for the treatment of Alzheimer’s disease. Furthermore, nanotechnology has been used extensively to overcome several pertinent issues like drug-resistance phenomenon, side effects of conventional drugs and targeted drug delivery issue in leprosy, tuberculosis and cancer. Thus, in this review, the application of different nanoparticles for the treatment of these four important diseases (Alzheimer’s disease, tuberculosis, leprosy and cancer) as well as for the effective delivery of drugs used in these diseases has been presented systematically. Although nanoformulations have many advantages over traditional therapeutics for treating these diseases, nanotoxicity is a major concern that has been discussed subsequently. Lastly, we have presented the promising future prospective of nanoparticles as alternative therapeutics. In that section, we have discussed about the futuristic approach(es) that could provide promising candidate(s) for the treatment of these four diseases.

## Introduction

Nanoparticles are particulate dispersions of solid particles ranging in size from 10 to 100 nm [1]. Nanoparticles have widespread applications in several disciplines including molecular biology, physics, organic and inorganic chemistry, medicine and material science due to their unique chemical, optical, electrical and magnetic characteristics compared with their bulk counterparts [2]. It has been found that reducing bulk materials to nano-size alters their physicochemical properties, which can be used in a variety of biomedical applications [3]. The large surface area-to-volume ratio of nanoparticles generally makes them highly reactive and confers a high adsorption capacity which in turn allows them to transport or interact with other molecules such as proteins, drugs, chemical compounds, etc [3]. Moreover, these interactions modulate the molecular or cellular activities, which make them a promising candidate for a variety of biological applications [3].

Drug delivery systems are one of the most promising areas in the field of health-care and biomedical research. Even though there has been a lot of advancement in the field of drug delivery systems, it is still a major challenge for scientists to find an effective carrier to deliver drug to the body that has a high benefit-to-risk ratio [4]. The use of drug delivery systems on a nanoscale is a significant step towards minimizing the chemotherapy-related adverse effects while simultaneously boosting the overall effectiveness of the treatment. While designing drug delivery systems, the utilization of nanomaterials offers unrivalled flexibility to tailor the inherent features of the therapeutics which include drug release characteristics, biodistribution, blood circulation half-life, cellular uptake, cell penetration, targeting and immunogenicity [5–7]. As numerous physiological processes occur at nanoscales, the comparable size of nanomaterials to the human cell organelles makes them a potent carrier for delivering various drug molecules [5–7]. Nanoparticles are favourable platforms for the target-specific and controlled delivery of therapeutic micro- and macromolecules due to their ability to form stable interactions with ligands, variability in size and shape, high carrier capacity and an ease of binding to hydrophilic as well as hydrophobic substances [5–7]. Additionally, the adverse effects and limited bioavailability of the existing drugs render nanoparticles as an effective drug delivery carrier [5–7]. The advantages of using nanoparticles as a drug delivery system includes: (i) easily adjustable particle size and surface properties of nanoparticles to provide passive as well as active drug targeting after parenteral injection, (ii) nanoparticles can regulate and maintain drug release during transport and at the site of localization, modifying the organ distribution and subsequent clearance of the drug to boost therapeutic efficacy and minimize adverse effects, (iii) controlled release and particle degradation can be easily altered by adjusting the matrix components of the nanoparticles; they also help in incorporating drugs in higher loads into the systems which is crucial for maintaining the drug activity, (iv) attaching targeted ligands to the surface of nanoparticles or employing magnetic guiding allows for site-specific targeting of the drug and (v) adaptability of the system to a wide range of administration methods including oral, nasal, parenteral, intra-ocular, etc.

Beside drug delivery, nanomaterials have been utilized in improving biomedical detection and imaging due to their unique passive, active and physical targeting properties [8–10]. The passive targeting strategies as well as the surface labelling strategies of nanoparticles with ligands which can target a specific receptor, and can often improve localization of imaging contrast agent in lesions [8–10]. For example, it has been shown that prostate-specific membrane antigen RNA aptamers on the surface of gold nanoparticles (AuNPs) offer a high computed tomography (CT) density for imaging prostate cancer cells [11]. Functionalized nanoparticles (conjugated with specific antibody) are often used in transmission electron microscopy (TEM) for achieving better contrasting images [12–14]. Surface plasmonic behaviour and fluorescence properties of nanoparticles enable us to acquire near infrared (NIR) transmission images and fluorescence correlation spectroscopic (FCS) images under different experimental conditions [15–17]. It has also been shown by different investigators that functionalized [dithiolated diethylenetriaminepentaacetic acid (DTDTPA)] or metal oxide (Fe_3_O_4_) embedded nanoparticles are better contrasting agents than conventional contrasting agents for magnetic resonance imaging (MRI) [18]. Furthermore, the surface of nano-sized superparamagnetic iron oxide (SPIO) agents coated with a high-affinity anti-EGFR antibody has been shown to target lung tumour by MRI [19]. Beside imaging purposes, inorganic-based nanoparticles are extensively used in developing different clinical diagnostic methods for detecting many important diseases such as cancer, tuberculosis, Alzheimer’s disease, etc [20,21]. Extensive research has been carried out worldwide to understand the therapeutic value of inorganic-based nanoparticles as anti-fungal, anti-viral and anti-bacterial agents [22,23]. These types of nanoparticles are also potential candidates for the effective treatment of various neurodegenerative diseases such as Parkinson’s disease, Alzheimer’s disease, infectious diseases (like tuberculosis and leprosy) and cancer [20,21,24,25]. Organic-based nanoparticles (liposomes, micelles and polymer-based nanoparticles) also have many therapeutic applications. These classes of nanoparticles are mostly used for gene therapy [26]. In fact, many organic nanoparticles based drugs have been approved by Food and Drug Administration (FDA) and European Medicines Agency (EMA) for clinical use. For example, liposomal nanoparticles framework containing compounds, Epaxal and Inflexal V, are used for hepatitis A and influenza, respectively [27,28]. Liposomal formulation of verteporfin can be effectively used to treat macular degeneration [29]. Drugs having organic nanoparticle based framework are also used for the treatment of cancer and fungal diseases [30,31]. Such diversified therapeutic applications of nanoparticles motivated us to frame this review. Here, we summarized the application of different nanoparticles for the treatment of four important diseases (Alzheimer’s disease, tuberculosis, leprosy and cancer) as well as the effective delivery of drugs used in these diseases. Subsequently, nanotoxicity and the promising future prospective have also been discussed.

## Nanotherapeutics: a promising tool for the treatment of Alzheimer’s disease

Protein fibrillation/aggregation has become a striking field in biomedical research for many decades owing to its association with major human disorders especially age-related dementia. Dementia comes in a variety of forms out of which Alzheimer’s disease (AD) is the most prevalent form [32], found in population preferentially aged 65 years or above. According to the World Alzheimer Report 2021, dementia is now the seventh leading cause of death worldwide. Hence dementia, particularly AD, has been considered as a global health priority [33]. Many neuropathologists have identified amyloidal plaques and neurofibrillary tangles (NFT) in the autopsied brain of people with AD, suggesting the involvement of these pathologies in the progression of the disease [34]. Amyloid plaques are extracellular deposits of fibrillar aggregates formed by amyloid-β peptide (Aβ). These plaques are the main component of the senile plaques found in the brain of patients [35].

Amyloid-β or Aβ peptide is a peptide composed of 35–42 amino acids [36], predominantly found in random coil conformation. This particular peptide is produced by a type I transmembrane amyloid precursor protein (APP). The processing of APP via the amyloidogenic pathway involves the enzymatic metabolism of APP to Aβ through the sequential cleaving of APP by two membrane-bound endoproteases, β- and γ-secretase [37,38] generating Aβ of variable C-terminal constitutions. Out of numerous different Aβ species, the peptides terminated at position 40 (Aβ_1-40_) and position 42 (Aβ_1-42_) are the most abundant (Aβ_1-40_): ∼80–90%, Aβ_1-42_: ∼5–10%). Aβ_1-42_ is more hydrophobic and fibrillogenic as compared with Aβ_1-40_ and is the principal species deposited in the brain [39].

Currently, Aβ peptide aggregation into toxic, prefibrillar oligomers are considered to be the key pathogenic event in the onset of AD [40]. Thus, blocking aggregation/fibrillation process of the concerned peptide (while sparing Aβ generation) is a preferred way to avoid mechanism-based toxicity. Several disease modifying approaches that includes small molecules, phytochemicals and nanoparticles have been employed [37] for the treatment of AD. Amongst all, nanoparticles are particularly intriguing because of several advantages that have been explained in the following section.

### Exploration of different nanoparticles for enhanced permeability and effective inhibition of Aβ fibrillation process

One of the biggest challenges of targeting therapeutics is the release of therapeutic candidates and their penetration through the BBB. This barrier precisely maintains the homeostasis of the brain and protects it from blood-borne toxic substances as well as microorganisms. Its structural architectures provide a tight structure and create a high trans-endothelial electrical restriction that plays a crucial role in maintaining the cellular microenvironment [41]. But this rigid structure also restricts the entry of a number of therapeutic candidates to the brain making them unsuitable for the treatment.

**Figure 1:**
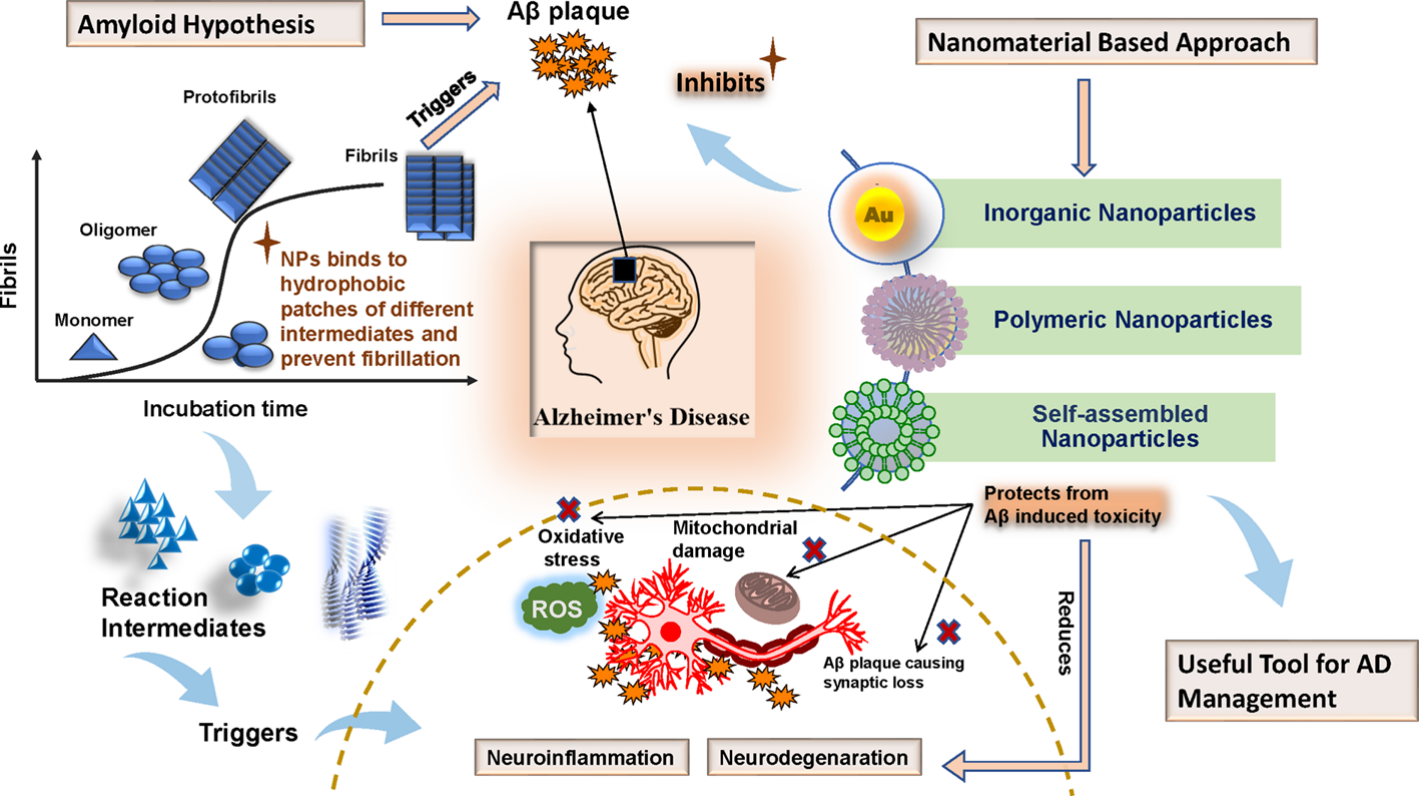
Schematic illustration of anti-amyloidogenic and cytoprotective effects exerted by metallic/inorganic, polymeric and self-assembled nanoparticles against *in vitro* and *in vivo* Aβ fibrillation process Both inorganic and polymeric nanoparticles share almost similar mode of action to ameliorate Aβ induced toxicity by binding to different intermediates (i.e. the different forms of Aβ species found during the fibrillation process such as monomeric species, oligomeric species etc.) of fibrillation process and by neutralizing their toxic effects. The plot of “Fibrils vs Incubation Time” has been reprinted (adopted) with permission from “Peptide and Protein Mimetics Inhibiting Amyloid β-Peptide Aggregation, Takahashi T, Mihara H., Acc Chem Res. 2008, 41(10),:1309–1318. doi: 10.1021/ar8000475. Copyright (2008) American Chemical Society”.

Basically, drug molecules with high molecular weight and non-lipophilic in nature are unable to get access into the central nervous system (CNS) and there is an insufficiency in traditional drug delivery systems for which myriads of investigations are being carried out in recent years to develop efficient drug delivery mechanisms that can successfully cross the BBB [42]. In present times, apart from small molecules and protein therapeutics, there is an increase in the number of studies that have been focusing on nanoparticles as potential inhibitors of amyloid aggregation. Nanoparticles are intriguing because they are able to cross the BBB at low concentrations and show a certain degree of specificity towards amyloid deposits depending on their composition [43]. In addition, what makes nanoparticles even more attractive is the feature of large surface-to-volume ratio which facilitates their binding to different forms of Aβ species found during the fibrillation process (Figure 1) such as monomeric species, oligomeric species and fibrils. Nanomaterials prevent the assembly of monomers and oligomers from forming fibrils and plaques through hydrophobic interactions or with specific ligands and can also induce conformational changes of monomeric species [44–49].

The major classes of nanoparticles used in AD therapeutics are polymeric and metallic/inorganic nanoparticles. Several inorganic/metallic nanoparticles provide good platform for the efficient inhibition of fibrillation process either in bare form or in conjugation with different drug molecules and therapeutic phytochemicals.

#### Metallic/inorganic nanoparticles

Metallic nanoparticles are intriguing because of their diverse shape, size and physico-chemical features which enables them to get attached with various chemical groups including ligands, antibodies, drugs, peptides etc. Several metallic nanoparticles such as AuNPs, AgNPs, FeONPs, SeNPs, Ce_2_O_3_NPs etc. Most of them has been reported as nanocarriers for targeted delivery of therapeutic agents and neurosensing/imaging. Metallic nanoparticles having magnetic behaviour are extensively used due to their biodegradable and biocompatible nature for diagnosis and treatment including magnetic hyperthermia treatments. Magnetic NPs enable magnetic delivery of drugs ideal for directed delivery to affected tissue under the action of a magnetic field. However, potential neurotoxicity associated with different metallic/inorganic nanoparticles such as oxidative stress, free radical formation, immune response, lysosomal dysfunction and cell necrosis is a matter of concern for many researchers [50].

However, several inorganic and metallic nanoparticles provide good platform for the efficient inhibition of fibrillation process either in bare form or in conjugation with different fibrillation inhibitors. The most widely used metallic nanoparticle owing to few unique features like inert behaviour, local surface plasmon resonance absorption, tuneable structural and chemical properties is gold nanoparticle (AuNP) [51–59]. Bare AuNPs and AuNPs modified with peptides or other therapeutic molecules are majorly used for the treatment of AD and has shown anti-fibrillation property [54]. Bastus et al. showed that a combination of heat and gold nanoparticles can dissolve the amyloid deposits locally and remotely [55]. Moore et al. studied the effect of electric charge and surface chemistry of gold nanospheres on the modulation of Aβ fibrillation. They used gold nanoparticles coated with citrate, CTAB (cetyltrimethylammonium bromide), PAA [poly(acrylic acid)] or PAH [polyelectrolytes poly(allylamine)hydrochloride] and showed that surface chemistry as well as size determines inhibition ability to a great extent and electric charge determines the morphological features of aggregates. Among all the coatings, PAA-coated nanoparticles (with a diameter of 18 nm and less) are non-toxic in nature and showed the highest inhibition of Aβ_1-40_ aggregation/fibrillation via a dynamic exchange between bulk solution and NP-localized peptide at a lower stoichiometric ratio of 1:2,000,000 with the peptide [56].

NIR absorbing Au nanocages developed by Shi et al., entraps a chelator that chelates metal ions like Cu^2+^ that induce amyloid fibrillation and oxidative stress. In this novel methodology, Au nanocages with human IgG as a pore blocker bound via redox as well as thermal-sensitive arylboronic ester bond to generate phenylboronic acid functionalized Au nanocages (AuNC-PBA) that entraps clioquinol and causes its oxidative stimuli-response controlled release to act at the target site. AuNC-PBA possesses an edge length of about 50 nm and a negative charge over the surface that confer the ability to cross the BBB. When Aβ aggregation increase the level of H_2_O_2_, the arylboronic ester interaction breaks which is also accompanied by NIR light induced local heating that release clioquinol and helps in the chelation of Cu^2+^ to dissolve amyloid-β plaques and inhibition of H_2_O_2_ production. By this mechanism, this nanoformulation also helped in increasing cell viability of pheochromocytoma cells (PC12) to approximately 70% in contrast to cells induced with 5 μM Aβ-Cu^2+^ toxicity that reduced the cell viability to 41% [57].

The resonating capability of gold nanoparticles upon the exposure to the light of specific energies, producing heat that can be used for photothermal therapy (PTT) to disintegrate amyloid deposits. In a study, penetratin peptide-modified poly(ethylene glycol)-stabilized gold nanostars (AuNS) further modified with ruthenium complex as luminescent probes has been synthesized. The conjugation of penetratin peptide with the AuNP enhances the permeability across the BBB. Additionally, the irregular morphology of the nanostars resulted a larger surface area and a high NIR absorption-scattering ratio. Aβ aggregation by the BCA protein assay in absence and presence of nanostars (10 and 20 μg/ml), showed an increase in soluble content of Aβ from 26% to 73% and 86%, respectively. Additionally, photothermal experiment (Ru@Pen@ PEG-AuNS−Aβ aggregates irradiated by 808 nm laser for 3 min) was performed by exploiting the NIR absorption property of AuNS. ThT fluorescence signal decreased more when Ru@Pen@ PEG-AuNS−Aβ aggregates were exposed to NIR than of non-irradiated Ru@Pen@ PEG-AuNS−Aβ aggregates. Possibly, the AuNS produce local heat upon laser irradiation that dissociate Aβ fibrils and destroy their amyloidogenic potential [58].

An excellent hybrid nanoparticle combining two peptide inhibitors/β-sheet breaker peptides (VVIA and LPFFD) has been designed by Xiong et al. First, a molecular hybrid has been developed by combining these two peptide inhibitors into a single sequence. Thereafter cysteine residues have been incorporated to the end parts of these short peptides/β-sheet breaker peptides (VVIA and LPFFD) which allows their conjugation onto gold nanoparticle surface via Au-S chemistry. This hybrid AuNPs significantly inhibited Aβ_1-42_ fibrillation process. Such inhibition mainly occurs due to hindrance in oligomerization process and reduction of β-structures and conversion to a higher proportion of random coils. The synergistic inhibition of these nanoparticles was greater as compared to single inhibitor sequence. Additionally, an increase in cell viability from 48% to 82% was achieved at a lower dosage of hybrid AuNPs (0.1 nmol/L of nanoparticles containing 40 nmol/L of inhibitor peptides) [59].

Similar to gold nanoparticles, silver nanoparticles can cause rapid dissolution of the fibrils. In a study, poly(vinyl) pyrrolidone (PVP)-stabilized negatively charged triangular silver nanoplates (AgTNPs) were found to be more effective than the PVP-stabilized silver nanospheres. The workers showed that when Aβ fibrils were treated with these AgTNPs under near infrared illumination, only 1 h was needed to dissolve the fibrils as compared with nanospheres that took roughly 70 h. Most effective NPs i.e. AgTNPs selectively bind to the positively charged residues present within the amino acid sequence of the peptide disrupted the fibril structure which led to dissolution of Aβ fibrils (as revealed from TEM and AFM studies) and thereby reduces cytotoxicity (as revealed from cell viability studies using SH-SY5Y and BE-(2)-C cells) [60].

Similarly, selenium nanoparticles have been demonstrated to be a useful therapeutic approach [46,61,62]. In a study, selenium nanoparticles were conjugated with two targeting peptides (LPFFD and TGN) and further conjugated with chitosan which produce ‘dual function’ selenium nanoparticles [46]. Those are L1T2-SeNPs, L1T1-SeNPs and L2T1-SeNPs where selenium nanoparticles having a 1:2, 1:1 and 2:1 concentration ratio of LPFFD to TGN, respectively. These SeNPs can serve two major functions; inhibition of Aβ aggregation and facilitates penetration through BBB. Among these three selenium nanoparticles, L1T1-SeNPs was most effective for inhibiting the aggregation process and reducing associated cytotoxicity in PC12 cells [46]. Synergistic effect of peptide and nanoparticle block the active site of fibril formation and reduce free monomeric peptide concentration, thus causing inhibition of Aβ_1-40_ aggregation via hydrophobic and electrostatic connection. Also these nanoparticles facilitates penetration through BBB, and reduced Aβ-induced apoptosis by preventing ROS generation [46].

Leblanc and coworkers studied the anti-fibrillation potency of dihydrolipoic acid (DHLA) capped CdSe/ZnS quantum dots of size approximately 2.5 nm. When these nanoparticles were mixed with amyloid β, they form conjugates with peptide leading to reduction in the fibrillation process which was studied using a fluorescence marker (Thioflavin T). Studies performed by using different analytical tools like TEM and AFM demonstrated that there is a noticeable change in morphology of fibrils conjugated to the quantum dots. Both the length and width of the fibrils were significantly altered [62].

Inorganic NPs having magnetic properties have also been utilized for the targeted delivery of different drug molecules and phytochemicals to the affected tissue. For example, a nano formulation has been made by a group of researchers that involves magnetic core-shell mesoporous silica nanomaterials as a carrier for the site directed delivery of quercetin, a polyphenolic phytochemical with known anti-fibrillation and anti-oxidant activity. This novel nanomaterial having a surface-modified monodispersed magnetite core, prepared using sol-gel process by taking tetraethoxysilane as a precursor. Subsequent evaluation of the biological activity of the nanoparticles revealed (i) relatively high entrapment efficiency and loading capacity of quercetin (70.35% and 14.51%, respectively), (ii) a high releasing percentage of 50.28% and (iii) increase in cellular viability of primary hippocampal neuronal cells up to 85.2%. When these NPs encapsulated quercetin, the stability and bioavailability of this phytochemical were greatly enhanced. The magnetic behaviour of these NPs enabled magnetic force guided on site release of quercetin which effectively inhibits the fibrillation process by binding to lower order species. Moreover, the antioxidant nature of quercetin trapped mesoporous silica nanomaterials minimized Aβ induced ROS generation. Further, surface-modification with a polymer polyethylene glycol 3000 was done. This modification improved blood circulation, half-life and reduced cytotoxicity [63]. Apart from these, the use of iron oxide nanoparticles as novel carriers for drug delivery for neurodegenerative diseases has also been reported in the literature [64].

#### Polymeric nanoparticles

Besides inorganic nanoparticles, polymeric nanoparticles have immense therpeutic values. These nanoparticles are gaining wide attention due to their faster biodegradability, relatively greater stability in physiological conditions and propensity to exhibit diverse surface functionality that allows them to act as excellent carriers of drug molecules in a relatively higher concentration [35]. These nanomaterials are very effective for intranasal administration and encapsulation of hydrophilic therapeutic agents [35,65]. Among polymeric nanomaterials, polysaccharide based nanogels such as chitosan and alginate, polyethylene glycol, poly-(lactic-coglycolic acid) are widely used. In recent times, all these along with other polymeric nanoparticles are utilized for the treatment of many important diseases including Aβ-induced Alzheimer’s disease. Polymeric nanoparticles are usually excellent carriers for uptake into brain due to their capability to access tight junctions of the BBB and their ability to prolong the drug release as well as to protect therapeutic agents against enzymatic degradation. Reports available in the literature clearly depicted that this class of nanoparticles can efficiently inhibit the fibrillation process of Aβ under *in vitro* and *in vivo* conditions [65–68].

For example, copolymeric NiPAM:BAM nanoparticles with a varied hydrophobicity were found to exert anti-fibrillation effect against Aβ [47]. These polymeric nanoparticles were synthesized from the copolymerization of *N*-isopropylacrylamide (NiPAM) and different proportions of N-*t*-butylacrylamide (BAM) and acrylic acid. The inhibitory effects of the synthesized nanoparticles were studied on the Aβ_1-42_ fibrillation process. Workers found that the nucleation step which is the initial stage of fibrillation process is mainly affected by these nanoparticles while the elongation step was unaffected. As a matter of fact, after nucleation phase is reached, the fibrillation process proceeded with the same rate both in the absence and presence of the nanoparticles. Analysis of the fibrillation kinetic data demonstrated that binding of the monomeric and prefibrillar oligomers of the peptide to the nanoparticles prevented fibrillation [47]. Similarly, in the study by another group, it was revealed that these same nanoparticles were able to show reduction in Aβ_1-42_ induced cytotoxicity as well as remarkable reduction in amyloid fibrillation. By employing a mechanistic model, it was proposed that an optimal negative charge capacity balances two opposite forces (namely, hydrophobic binding and electrostatic repulsion) which are mainly responsible for the interaction between Aβ_1-42_ and the nanoparticles. ‘Aβ_1-42_-copolymeric NPs interaction’ further leads to the stretching of Aβ_1-42_ molecules thereby avoiding the formation of fibrillogenic β-sheet structure [48].

Brezesinski and co-workers have developed fluorinated polymeric nanoparticles by the complexation of a polyampholyte poly(N,N-diallyl-N,N-dimethylammonium-altmaleamic carboxylate) with alternating anionic and cationic monomers and perfluorododecanoic acid. The hydrodynamic diameter of these nanoparticles were 3-5 nm, small enough to cross the BBB. Usually, fluorinating compounds are helix inducers and mediate β-sheet to α-helix transition. It was shown from atomic force microscopy (AFM), immunoblot, and SDS-PAGE studies that there was a delay in the oligomerization of Aβ_1-40_ because of the transition (β-sheet to α-helix) caused by the fluorinated nanoparticles and was reduction in peptide-induced cytotoxicity by targeting the caspase-3 in SH-SY5Y cell line [69].

Recently, ubiquinone-10 or coenzyme Q10 loaded nanoparticles have been formulated from TMC (trimethylated chitosan) surface-modified PLGA [poly (D,L-lactide-co-glycolide)] nanoparticles [33]. The major advantages of these nano-coatings are: (i) they are biodegradable; (ii) they approved by FDA; and (iii) they can easily penetrate through the BBB. These nanoparticles possessed a mean diameter of 99.6 nm and zeta potential of −18.3 mV. The neuroprotective effects of Co-Q10-loaded nanoparticles were demonstrated by a reduced staining of senile plaque which is accompanied by the reduction in the Aβ concentration as well as inhibition of Aβ fibrils/aggregates. Apart from this, results obtained from various behavioural testing showed a significant improvement in memory impairment. These outcomes suggest Co-Q10 coated nanoparticles could efficiently dissolve the amyloid plaque formed by Aβ fibrils and can be an efficient disease modifying therapy against AD [33].

Polymeric nanoparticle with conjugated phytochemicals has gained wide attention in last few decades. A well-known phytochemical with good antioxidant and anti-amyloidogenic property often reported to prevent amyloid β fibrillation is resveratrol [33,70]. A couple of studies have been done to assess these properties using *in vitro* studies where its efficacy is evident. But resveratrol imparts minimal effect under *in vivo* conditions, because of three possible reasons: (i) its sparse concentration, (ii) short half-life time in plasma and (iii) rapid metabolism to glucuronic and sulfate metabolites [33,70]. To overcome this, resveratrol tagged nanoparticles have been prepared using poly-caprolactone (PCL) as the hydrophobic core and polyethylene glycol (PEG) as the hydrophilic shell. When these nanoparticles administered to PC12 cells equivalent to 10 μM of resveratrol, better cytoprotective effect was observed as compared with free resveratrol by neutralizing the toxic effect of the amyloid beta peptide [70].

Few researchers have tagged polymeric nanoparticles to certain phytochemicals along with a carrier protein ApoE (apolipoprotein E). A study reported that ApoE3 mediated stable poly(butylcyanoacrylate)(PBCA) nanoparticles containing curcumin (ApoE3-CPBCA) has been formulated to enhance cellular uptake and anti-amyloidogenic activity of the tagged compounds [71]. Cytotoxicity studies on SH-SY5Y neuroblastoma cells and anti-apoptotic activity studies suggested the higher efficacy and increased uptake of curcumin by ApoE3-C-PBCA as compared with curcumin not conjugated with any nanoparticles. These nanoparticles imparted excellent neutralization effect against Aβ-induced cytotoxicity due to the synergistic effect of ApoE3 and curcumin [33].

Similar to phytochemicals, several peptide molecules have the potential to inhibit the Aβ induced toxicity effectively. However, their hydrophilic nature restricts them to cross the BBB [33]. In this regard, nanotechnological intervention specifically polymeric nanoparticles based approach provides a useful targeting strategy to overcome the obstacle. For example, Agyare et al. developed such type of nanoparticles that enclosed the sub-fragments of Aβ peptide in a tripolyphosphate gelation modified polymeric chitosan. It was further coated with polyamine modified F(ab′) portion of antibody IgG4.1 which is an anti-amyloid antibody. This engineered nanoparticles were able to cross the BBB and targeted amyloid formed by the aggregation of the causative peptide [72]. Thus, polymeric nanoparticles are excellent tools for the delivery of therapeutic molecules that otherwise would not have gained entry through the BBB. Several polymeric substances have been described to increase the circulation time of tagged therapeutic molecules substantially and protect nanoparticles from opsonization process by acting as a steric barrier. The muco-adhesive features and highly functionalized structure of polymeric substances helps in the site directed sustainable release of the drug molecules to the affected site of brain. The biodegradability nature of this class of nanomaterials is the major attraction for many researchers. Upon degradation, they produce metabolites that could be easily processed through biochemical pathways occurring inside the body. However, one disadvantage of polymeric nanoparticles is they sometimes release the tagged therapeutic molecules in sites other than the targeted area as a result of which only empty polymeric vector is delivered to the affected site. In contrast, various metallic/inorganic nanoparticles themselves possess the anti-aggregation properties and effectively inhibit aggregation/fibrillation process. Like polymeric nanoparticles metallic nanoparticles can also be tagged with different chemical groups such as ligands, antibodies, peptides etc. Their unique structural features help them to get combined with other treatment methods for fibril dissolution such as magnetic hyperthermia treatment, photothermal therapies etc. Interestingly, the mode of action(s) exerted by metallic/inorganic nanoparticles towards the inhibition of *in vitro* and *in vivo* Aβ fibrillation process is/are almost similar to that exerted by different polymeric nanoparticles (Figure 1). While inorganic nanoparticles are excellent in preventing amyloid fibrillation, polymeric nanoparticles are more efficient in dissolving formed fibrils. Despite the advantages, several *in vitro* and *in vivo* studies have reported excessive production of ROS upon exposure to metallic nanoparticles. Production of ROS increases the oxidative stress, inflammation and thereby causing damage to several vital structures of the body such as DNA, proteins and cell membranes [73]. Hence, right selection of nanoparticles is the determining factor for the effectiveness of the nanomaterial-based therapeutics.

#### Self-assembled nanoparticles

Various other nanoparticles are also studied for their therapeutic activity against amyloidal aggregation such as self-assembled nanoparticles. Taylor et al. prepared click-curcumin liposomes by attaching the curcumin derivative [N-propargyl 2-(3′,5′-di(4-hydroxy-3-metoxystyryl)-1H-pyrazol-1′-yl)-acetamide] on preformed liposomes by click chemistry in PBS (pH 6.5). The mean diameter of these self-assembled nanoparticles varies from 52.8 to 218 nm. When tested for the fibrillation inhibition potential by ThT fluorescence assay and immunoassay, a substantial inhibition was observed in both oligomer and fibril formation. This is may be due to two possible factors. Firstly, due to the high affinity binding involving multivalent interactions between Aβ and click-curcumin liposomes. Secondly, because of the protrusion of curcumin-derivative molecule from the liposome surface, they are more accessible for the interaction with the Aβ species [74].

In another study, the amphiphilic nature of the N-terminal peptide fragment pN1-22 of ovalbumin is exploited and nanoparticles are synthesized by the self-assembly process in PBS at a temperature of 65°C and pH 2.2. Additionally, these nanoparticles are noncytotoxic as evident from cell viability assay performed on PC12 cells that showed 100% viability at a concentration of 46 μM. The resulting nanoparticles possessed a diameter of around 30 nm and ζ-potential value of 35 mV. ThT fluorescence assays and morphological studies by TEM imaging showed the concentration dependent inhibition of Aβ_1-42_ fibrillation in presence of pN1-22 nanoparticles. The half time (*t*_1/2_) of Aβ fibrillation was increased with increasing concentration of the NPs that indicates the pN1-22 nanoparticles prevented Aβ fibrillation by binding to Aβ monomer as well as oligomers majorly through hydrophobic interaction and hydrogen bonding [49].

Li et al., reported self-assembled nanoparticles based novel two-in-one strategy of real time assessing as well as inhibition of Aβ peptide. Initially, the group self-assembled Aβ 15-20 peptide (KLVFF) and polyoxometalate (POM) which yielded hybrid colloidal nanospheres having the diameter ranging from 70 to 100 nm. Subsequently, they incorporated Congo red (a clinically used amyloid β fibril specific staining dye) into this hybrid colloidal nanosphere so as to monitor the fibrillation inhibition process via change in Congo red fluorescence. The synthesized nanomaterials possess combined anti-aggregation property of the β-sheet breaker peptide and POMs in a single system that resulted in higher inhibitory potential. At a 2:1 concentration ratio of Aβ_1-40_ and the self-assembled POM-peptide nanospheres, the fibril bound ThT fluorescence was reduced greatly (65%). Whereas the same was reduced by 45% and 35% in presence of identical amount of peptide (KLVFF) and POM, respectively. In addition to this, the nanoparticles conferred targeted inhibition of Aβ aggregation in mice cerebrospinal fluid and reduced Aβ induced cytotoxicity [75].

Recent advances in the field of nanoquencher based biosensing has guided many researchers to formulate novel biosensing based inhibitors. Xia and co-workers prepared a nanosystem where self-assembled polydopamine nanospheres (PDANS), a nanoquencher, were conjugated with a carboxyfluorescein (FAM)-labelled DNA aptamer fluorophore. This nanosystem is useful for selective detection of Aβ oligomers via ‘fluorescence-signal on’ strategy. The mechanism behind this detection strategy involves the specific interaction of Aβ oligomers with FAM-DNA aptamers causing the fluorophore to change its conformation into a hairpin structure and is subsequently released from the surface of the nanoquencher PDANS that leads to the increase in the FAM-DNA fluorescence signal. A detection limit of 12.5 nM was achieved by this strategy. Further, the fibrillation inhibition potential of PDANS was also observed because of its interaction with Aβ monomeric species via hydrogen bonding [76]. This nanosystem could be an efficient diagnostic tool for detection of Aβ oligomers associated amyloid beta induced Alzheimer's disease.

At present, the implementation of nanotechnology-based therapeutics has gained tremendous consideration because of their ability to penetrate through the BBB and being able to safeguard the native structure of the inhibitor molecules. Several metallic/inorganic, polymeric as well as self-assembled nanoparticles conjugated with well-established inhibitors (phytochemicals, enzymes) have been designed as dual functional nanoparticles that confer the ability to get penetrated through BBB and have efficient anti-Aβ activities (summarized in Table 1). Additionally, many metallic nanoparticles are also being employed to treat several infectious diseases like tuberculosis and leprosy.

**Table 1:** Potential usage of nanoparticles (NPs) against amyloid β fibrillation induced Alzheimer’s disease

Class of NPs	Various NPs in each class	Brief description	Doses	Mode of action/drug release	Nature of studies	References
Inorganic	Gold nanospheres of varied surface coatings and size (8, 18 and 40 nm)	Gold nanoparticles coated with citrate, CTAB (cetyltrimethylammonium bromide), PAA [poly (acrylic acid)] or PAH [polyelectrolytes poly(allylamine)hydrochloride] found to alter the extent of fibrillation differently at a concentration range of Pico molar scale.	Highest inhibition in a lower stoichiometric ratio of 1:2,000,000	These nanoparticles are able to cross BBB and easily undergo clearance. 18 nm non-toxic PAA-coated NPs showed complete inhibition of Aβ_1-40_ aggregation via a dynamic exchange between bulk solution and NP-localized peptide.	*In vitro* and cell culture study	[56]
	NIR absorbing gold nanocages	Au nanocages with human IgG as a pore blocker bound via redox as well as thermal-sensitive arylboronic ester bond to generate phenylboronic acid functionalized Au nanocages entrapping a chelator, clioquinol that chelates Cu^2+^ known to induce amyloid fibrillation and oxidative stress. Edge length: 50 nm	Cell viability of PC12 cells increased to 70% in contrast with 5 μm Aβ_1-40_ at a dose of 0.5 mg/ml	When Aβ_1-40_ aggregation increase the level of H_2_O_2_, the arylboronic ester interaction breaks (also accompanied by NIR light induced local heating) thus releasing clioquinol to chelate Cu^2+^ and to dissolve amyloid-β plaque. Furthermore, inhibit aggregation induced H_2_O_2_ production and thus improved cell viability.	*In vitro* and cell culture study	[57]
	Penetratin peptide-modified poly(ethylene glycol)-stabilized gold nanostars (AuNS)	Penetratin peptide-modified poly(ethylene glycol)-stabilized AuNS further modified with ruthenium complex (Ru@Pen@ PEG-AuNS) as luminescent probes; synthesized using seed mediated growth method; ability to cross the BBB, advantage of tracking drug delivery. Size: 105 nm	Increased soluble content of Aβ from 26% (without NPs) to 86% in presence of 20 μg/ml Ru@Pen@ PEG-AuNS	Irregular morphology of the AuNS resulted larger surface area and high NIR absorption-scattering ratio; local heat upon laser irradiation dissociates Aβ fibrils and destroy their amyloidogenic potential thereby increase soluble Aβ content.	*In vitro* cell culture and animal studies	[58]
	β-Sheet breaker peptides conjugated gold nanoparticles	Molecular hybrids of a combination of two peptide inhibitors (VVIA and LPFFD) into single sequences (VVIACLPFFD) and conjugated onto gold nanoparticles (AuNPs) by modified Frens method. Size: 15 nm	0.1 nmol/L of NPs containing 40 nmol/L of inhibitor peptides	Special surface orientation and synergetic interactions between NPs and Aβ cause strong inhibitions of Aβ_1-42_ oligomerization and fibrillation by inducing higher proportion of random coils; increases cell viability from 48% to 82% in SH-SY5Y cells.	*In vitro* and cell culture study	[59]
	poly(vinyl) pyrrolidone (PVP)-stabilized negatively charged silver nanoparticles	PVP-stabilized negatively charged triangular silver nanoplates (AgTNPs) shown to be more effective than the silver nanospheres and dissolve fibrils in only 1 h upon near infrared irradiation. Nanospheres Edge length of nanoplates: 70 nm and diameter of nanospheres: 20 nm Increase in cell viability: 65%	30 nM administered into SH-SY5Y and BE-(2)-C cells	AgTNPs selectively binds to the positively charged residues present within the amino acid sequence of the peptide disrupt the fibril structure and led to dissolution. The larger surface area of nanoplates adsorbs greater number of Aβ monomers.	*In vitro* and cell culture study	[60]
	Selenium nanoparticles	Selenium NPs conjugated with two targeting peptides (LPFFD and TGN), further conjugated to chitosan act as “dual function” selenium nanoparticles; prepared in presence of ascorbic acid; peptides taken in ratios of 1:2, 1:1 and 2:1, and the resultant NPs termed L1T2-SeNPs, L1T1-SeNPs and L2T1-SeNPs; L1T1-SeNPs shows highest aggregation inhibition and reduced cytotoxicity in PC12 cell. Size: around 100 nm	0.5 mg/kg	Synergistic effect of peptide and nanoparticle block the active site of fibril formation and reduce free monomeric peptide concentration thus, causing inhibition of Aβ_1-40_ aggregation via hydrophobic and electrostatic connection. Also facilitates penetration through BBB; reduced Aβ induced apoptosis by preventing ROS generation.	*In vitro* cell culture and animal studies	[46]
	CdSe/ZnS quantum dots	Dihydrolipoic acid (DHLA) used as a capping agent. size: 2.5 nm	1.4 μM (for TEM studies)	Nanoparticles interact with amyloid β, to form conjugates leading to change in morphology of fibrils and reduced fibrillation.	*In vitro*	[62]
	Novel hybrid systems of magnetic core-shell mesoporous silica NP and quercetin.	Synthesized by sol-gel process in the presence of a precursor tetraethoxysilane and modification with PEG3K for reduced cytotoxicity; relatively high entrapment efficiency and loading capacity of quercetin, increase in cellular viability.	10 and 20 μM NPs administered into primary hippocampal neuronal cells	Increased stability, bioavailability of quercetin; magnetically directed on site release of quercetin; leads to better inhibition of the fibrillation process by binding to lower order species; minimization of Aβ induced ROS generation.	Cell culture studies	[63]
Polymeric	Copolymeric nanoparticle	Copolymerization of *N*-isopropylacrylamide (NiPAM) and different proportions of N-*t*-butylacrylamide (BAM) done by free radical polymerization in presence of SDS. Particle size: 40 nm	–	Nanoparticles bind with the monomeric and prefibrillar oligomers and prevent Aβ_1-40_ fibrillation by blocking nucleation step. No effects on elongation step; fibril modulating effect depends on the capacity of the negative surface charges carried by these NPs.	*In vitro*	[47,48]
	Fluorinated polymeric nanoparticle	Composed of polyampholyte poly (N,N-diallyl-N,N-dimethylammonium-altmaleamic carboxylate) and perfluorododecanoic acid by free radical polymerization. Hydrodynamic diameter: 3–5 nm	Cell tolerance upto 100 μg of NPs	Delayed oligomerization of Aβ_1-42_ due to β-sheet to α-helix transition caused by fluorinated nanoparticles. Reduced cytotoxicity by targeting caspase-3 in SH-SY5Y cell line.	*In vitro* and cell culture studies	[69]
	Ubiquinone-10- trimethylated chitosan-modified-poly (D,L-lactide-co-glycolide) PLGA nanoparticles	Ubiquinone-10 or coenzyme Q10 loaded nanoparticles formulated from trimethylated chitosan (TMC) by nanoprecipitation method; TMC surface-modified with PLGA nanoparticles; biodegradable and can easily penetrate through the BBB. Diameter: 99.6 nm	-	Neuroprotective effects of Co-Q10-loaded NPs evident from reduced staining of senile plaque accompanied by the reduction in the Aβ concentration as well as inhibition of Aβ fibrils/aggregates.	*In vivo* (Mouse model)	[33]
	Resveratrol tagged polymeric nanoparticle	Resveratrol tagged nanoparticles have been prepared using poly-caprolactone (PCL)-polyethylene glycol (PEG) NPs by nano-precipitation method; Diameter: 70 nm	Nanoparticles administered to PC12 cells equivalent to 10 μM of resveratrol	Protects from amyloid β-induced damage in a dose dependent manner; attenuates intracellular oxidative stress and caspase-3 activity.	*In vitro/in vivo*	[70]
	ApoE3 mediated stable poly(butylcyanoacrylate) (PBCA) nanoparticles containing curcumin (ApoE3-CPBCA)	Curcumin tagged polymeric nanoparticles prepared by an anionic polymerization method. Particle size: 1972.3 nm	Nanoparticles administered to SH-SY5Y cells equivalent to 50 and 100 nM of curcumin	Enhance cellular uptake and anti-amyloidogenic activity of the tagged compounds by receptor mediated endocytosis; excellent neutralization effect against Aβ-induced cytotoxicity due to the synergistic effect of ApoE3 and curcumin.	*In vitro* and cell culture studies	[71]
	Aβ sub-fragment tagged chitosan nanoparticle synthesized by ionotropic gelation method	Aβ sub-fragment enclosed in a tripolyphosphate gelation modified polymeric chitosan (at different concentration) further coated with polyamine modified F(ab′) portion of anti-amyloid antibody IgG4.1; preparation method: ionotropic gelation; able to cross the BBB. Polymer core size: 250 nm	Not applicable	Useful in developing delivering diagnostic or therapeutic agents across the BBB that can specifically target amyloid deposits.	*In vitro/in vivo*	[72]
Self- assembled	Ovalbumin (OVA)-derived amphiphilic peptide based nanoparticle	Self-assembled N-terminal peptide of OVA (pN1-22) prepared by the self-assembly process in PBS at a temperature of 65°C and pH 2.2. Diameter: 30 nm	46 μm NPs administered to PC12 cells	Monomeric and/or oligomeric Aβ_1-42_ bind to the NPs via H-bonding and hydrophobic interaction; decrease the solution concentration of Aβ and delays aggregation.	*In vitro*	[49]
	poly(dopamine) NPs	Self-assembled poly(dopamine) NPs constructed in an alkaline medium in presence of oxygen as the oxidant; selectively detect Aβ oligomers with a concentration as low as 20 nm with the help of carboxyfluorescein (FAM)-labelled DNA aptamers-polydopamine nanospheres (PDANS)-conjugated NPs; high selectivity. Average diameter: ∼80 nm	Not applicable	A two-in-one system; efficiently retard the fibrillation by interacting with Aβ via hydrogen bonding; conformational change of the DNA aptamer bound to Aβ_1_-_42_ oligomer, causes its release from the nano quencher PDANS there aiding in the detection of Aβ oligomers by “signal on” fluorescence strategy.	*In vitro*	[76]
	Self-assembled hybrid NPs of polyoxometalate (POM)-peptides and Aβ_15_-_20_ peptides	Colloidal nanospheres of POMs and β-sheet breaker peptide Aβ_15-20_ exert their combinatorial anti-aggregation properties in one system; attached Aβ fibril specific Congo red dye serves excellent fluorescent probe for real-time screening; good stability in physiological condition (PBS and CSF) Preparation method: self-assembly approach. Diameter: 80 nm	6 μM	This two-in-one bifunctional nanoparticles can simultaneously bind to the homologous sequence in Aβ peptides and inhibit their aggregation and increase the targeting inhibition efficacy; Also increased PC12 cell viability to 82%.	*In vitro/in vivo*	[75]
	Curcumin derivative-conjugated liposome-nanoparticles	Curcumin-modified liposome synthesized using click chemistry and thin film hydration method with high affinity binding with Aβ. Diameter range: 52.8 nm to 218 nm		Efficiently binds to Aβ via multiple interactions with Aβ and inhibits both fibrillation and oligomer formation in concentration dependent manner; protrusion of curcumin-derivative molecules from the liposome surface are more accessible for the interaction.	*In vitro*	[74]

## Nanotechnology based strategies to treat tuberculosis

### Tuberculosis and drug resistance

Tuberculosis (TB) is referred as an immemorial human malady and the discovered etiological agent was *Mycobacterium tuberculosis* [77]. According to the WHO report 2021, approximately 4.8 million people were diagnosed with pulmonary TB in 2020 globally. For efficacious treatment of tuberculosis, a six-month course of drug is administered to the TB diseased patients. The four first-line drugs: isoniazid (INH), rifampicin (RF), ethambutol (EMB) and pyrazinamide (PYZ) are basically used [78]. In spite of development of various effective anti-TB drugs, TB is still considered as a dreaded disease which affects the mankind with highest mortality and morbidity worldwide [79]. The drug resistance phenomenon is considered as the major obstruction in the proper treatment of TB. Consequently, in patients who developed rifampicin-resistant TB (RR-TB) and multidrug-resistant TB (MDR-TB, where the resistance is developed towards isoniazid and rifampicin, the two most effective anti-TB drugs), the treatment is prolonged, expensive with several side effects as well [78]. Apart from this, pre- XDR TB and XDR TB are of major concern. Pre- XDR TB is a diseased condition where the patient is resistant to rifampicin and any fluoroquinolone, a class of second-line anti-TB drug and XDR TB is a type of diseased condition where the patient is resistant not only to rifampicin and any fluoroquinolone, but also to at least one of the drugs i.e., bedaquiline and linezolid. The universally used vaccine for TB is bacille Calmette-Guerin (BCG) vaccine. This vaccine consists of live attenuated strain of *Mycobacterium bovis*. It has achieved success in prevention of TB in children but is inefficient to prevent the disease in adult individuals [80]. To overcome the problem of drug resistance, lack of effective vaccine and to achieve the End TB Strategy targets for 2030 and 2035, there is an urgent need of adequate research and innovation in this regard [78].

### The use of nanoparticles for the treatment of tuberculosis

Mycobacteria are blessed to have a critical system of survival, which consequently help them to exist in the hostile environment of the host. Furthermore, the patient incompliance and the drug resistance phenomenon (both multidrug-resistance, MDR/extensively drug-resistance, XDR) adds a bit more complexity for the treatment of tuberculosis. The current approach of nanotechnology involves greater prospect to be utilized for the treatment of TB, by preparing different nanoformulations which is yet to be explored [81]. The primacy of this therapeutic approach have several features: (i) efficient carrying capacity, (ii) more time span activity, (iii) facile route of administrations, (iv) loading of various drugs or easy encapsulations and (v) lower toxicity towards the host cells [82].

Myriad of metallic nanoparticles have been employed as anti-mycobacterial agents for several years [83]. Among them, silver and gold nanoparticles (AgNPs and AuNPs) are considered as important and effective because of their distinctive physical, chemical and biological characteristics [84]. In former times, silver was considered as an essential antimicrobial agent in treatment of various infectious entities [85]. So, both of these AgNPs and AuNPs may exhibit antimycobacterial effect alone or by being conjugated with various stabilizing agents/antibiotics/peptides/polymers to show a combinatorial effect which will help in the treatment of MDR/XDR TB [84]. It has been shown that these two nanoparticles can inhibit the growth of bacillus Calmette-Guerin (BCG), which is used as a surrogate for TB. Liang and co-workers used tdTomato, an important fluorescent protein expressed by mycobacteria in culture, so as to determine the number of viable mycobacterial cells by examining the fluorescence intensity. It was observed that the BCG fluorescence had decreased maximally upon the treatment with lower concentrations of citrate capped AuNPs (0.1 and 1 μg/ml) and higher concentrations of polyallylamine hydrochloride (PAH) capped AuNPs (10 and 20 μg/ml). On the other hand, the extent of inhibition of BCG fluorescence was almost similar in presence of 1–20 µg/ml AgNPs. The alterations in the number of colony forming units (CFUs) observed after the treatment with these nanoparticles were in agreement with the results of fluorescence experiment [86]. An independent study by Ignatov and coworkers revealed that the growth of *M. tuberculosis* H37Rv was inhibited in the *in vitro* treatment of 0.1–50 μg/ml polyvinylpyrrolidone (PVP) stabilized single dispersed silver nanoparticles (SNPs-PVP) [85]. The maximum inhibition (by two times) was observed in presence of 50 µg/ml SNPs-PVP. Similar results had also obtained in *in vivo* studies with experimental mouse model. The mycobacterial load in lungs and spleen of the mice had decreased by two times due to the SNPs-PVP treatment (0.1 mg/kg). Additionally, the effect of the treatment was also examined for inflammatory phenomenon in the lungs and the survival rate of the mice. Inflammation in lungs were decreased substantially and the survival rate had risen up to 60%. The interleukin-4 level (IL- 4; an anti-inflammatory cytokine), in the broncho- pulmonary lavage fluid (BPL) after the SNPs-PVP treatment was reduced by two fold which possibly led to the decrease in inflammatory phenomenon in the lungs of experimental animals. SNPs-PVP treatment additionally reduced the level of different immunological markers such as interferon-γ (IFN-γ), tumour necrosis factor-α (TNF-α) etc. in body fluids of experimental TB mice. By considering all the results obtained from both the *in vitro* and *in vivo* studies, it can be concluded that SNPs-PVP can be considered as effective therapeutic agents against TB [85].

Attempts have also been made to study the inhibitory effect of silver nanoparticles towards drug resistant (both MDR and XDR) *M. tuberculosis* strains. Chen et al. explored the anti-mycobacterial activity of synthesized AgNPs [where alginate (ALG) was utilized as a capping agent] against numerous pathogenic mycobacterial strains which includes both drug-sensitive and drug-resistant strains (MDR and XDR strains) [81]. It was concluded from this study that alginate capped silver nanoparticles (ALG-AgNPs) inhibited the growth of above mentioned mycobacterial strains, without conferring any toxicity to the THP1 cells. In another approach of this study, when latency was induced *in vitro*, it had been observed that the growth of *M. tuberculosis* H37Rv was getting reduced appreciably in a dose-dependent manner of ALG-AgNPs, which implied that these nanoparticles can also be used to treat latent TB by subduing the dormant *M. tuberculosis*. Fluorescence-based cell permeability experiment revealed that ALG-AgNPs treatment increases the cell permeability which may be the possible mechanism behind its anti-mycobacterial activity. The therapeutic effect of ALG-AgNPs was further assessed by executing the *in vivo* studies on zebrafish and mouse TB model which unveiled that the bacterial load was reduced upon the treatment of these nanoparticles without causing any cytotoxicity in the host cell [81]. Even, silver containing mesoporous silica-based nanosystems (MSNs-AgBrNPs and Ag@MSNs) have growth inhibitory effects [87]. Interestingly, MSNs-AgBrNPs had shown efficient antimycobacterial effect in comparison with Ag@MSNs. The possible mechanism of better efficiency of MSNs-AgBrNPs over Ag@MSNs could be due to the distribution of AgBrNPs throughout the silica network and mesoporous channels; thus more exposed to the media, in comparison with the Ag core present in Ag@MSNs. The morphological studies (carried out by cryo-EM) further revealed that MSNs-AgBrNPs exerted efficient anti-mycobacterial activity by damaging the cellular envelope of *M. tuberculosis* H37Rv. Furthermore, in this paper, authors hypothesized that various drug candidates can also be loaded with these mesoporous nanosystems which can be considered as a better therapeutic approach for the treatment against drug resistant (both MDR and XDR) *M. tuberculosis* strains [87].

The anti-mycobacterial activities of biosynthesized AuNPs, AgNPs, and Au-AgNPs (synthesized from leaf extract of *B. prionitis*, root extract of *P. zeylanica* and bark extract of *S. cumini*) have also been explored. A study by Singh et al. depicted the growth inhibitory effect of these nanoparticles against *M. tuberculosis* in latent and active disease conditions [88]. The bimetallic nanoparticles i.e. Au-AgNPs are much more efficient in context of anti-mycobacterial activities than the monometallic ones (AuNPs and AgNPs). This study also revealed that Au-AgNPs synthesized from the bark extract of *S. cumini* plant, exhibit higher specificity and selectivity to the pathogen *M. tuberculosis*. Therefore, it can be concluded that the bimetallic nanoparticles i.e. Au-AgNPs could be more promising drug candidate than monometallic nanoparticles (AuNPs and AgNPs) for the treatment of tuberculosis [88].

Silver nanoparticles were found to enhance the cell membrane permeability by generating nano-sized pores on it. This consequently might be helpful for the entry of antibiotics [89]. It had also been examined that by encapsulating silver to a biocompatible polymer (e.g. poly (D,L-lactide-co-glycolide); PLGA), effective antibiotics can be delivered directly to the infected area of alveolar macrophages [83].

**Figure 2:**
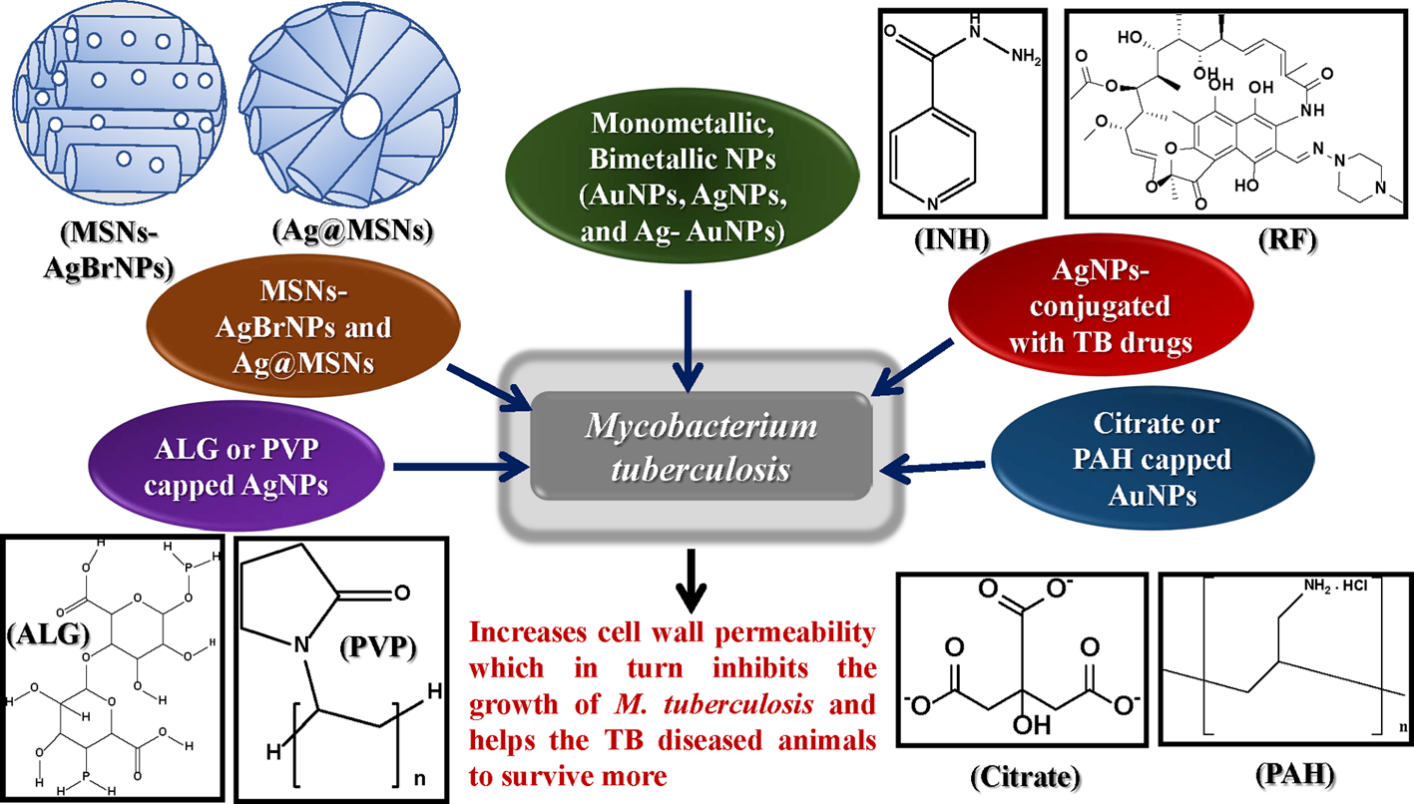
Impact of various metallic nanoparticles in tuberculosis Consolidated elucidation of various effects of cell penetrable bare/capped monometallic (AgNPs, AuNPs, citrate capped AuNPs, PAH capped AuNPs, ALG capped AgNPs, PVP capped AgNPs etc.) and bimetallic nanoparticles (Ag-AuNPs) on *Mycobacterium tuberculosis* pathogen. ALG, alginate; INH, isoniazid; PAH, polyallylamine hydrochloride; PVP, polyvinylpyrrolidone; RF, rifampicin; MSNs-AgBrNPs and Ag@MSNs, silver-containing mesoporous silica-based nanosystems. Image of MSNs-AgBrNPs and Ag@MSNs have been redrawn (adopted) from “Mesoporous silica nanoparticles containing silver as novel antimycobacterial agents against *Mycobacterium tuberculosis*, 197, Montalvo-Quirós S, Gómez-Graña S, Vallet-Regí M, Prados-Rosales RC, González B and Luque-Garcia JL, 111405, Copyright (2021) with permission from Elsevier”.

Interestingly, it has been observed that the anti-mycobacterial activity of gold nanosystems [gold nanorods (AuNRs) and gold nanospheres (AuNSs)] can be improved upon their administration with different conventional TB drugs. In a study by Ali et al., a drug carrier system had been prepared where AuNRs and AuNSs were conjugated with rifampicin (RF) which was meant to be delivered to the macrophage cells after the engulfment, and has shown anti-TB effect. It can be concluded from this study that these gold nanosystems could be considered as highly efficient drug carrier system in the therapeutic approach towards tuberculosis [90]. Other investigators also examined whether silver nanoparticles are able to carry drugs which are usually used for the TB treatment. Sun et al. revealed that the AgNP-Vancomycin (AgNP-VAM) conjugates which were tested on *Mycobacterium smegmatis*, showed significant anti-mycobacterial effects [91]. It has been found that by conjugating with AgNPs, the uptake of VAM was significantly enhanced, resulting in proper delivery of the antibiotic into the bacterium. By considering these results, it can be concluded that the current approach can be utilized for a better delivery of drug for the treatment of TB [91]. Another independent study by Golikov and coworkers, it was revealed that the inhibition of growth of mycobacteria was maximum with the usage of silver nanoparticles along with different drugs (isoniazid, rifampicin, ethionamide, levofloxacin and ofloxacin) and minimum when AgNPs were used along with kanamycin [92]. Similar results had also been observed in case of *in vivo* studies performed by this group. The survival rate of mice treated with silver nanoparticles with isoniazid were maximum i.e. ∼95%. While, mice treated only with the silver nanoparticles, the survival rate was found to be ∼35%. From these studies, it can be concluded that silver nanoparticles in combination with different antibiotics could be used as a promising therapy for the treatment of TB [92]. The results obtained have paved the way for the possibilities of use of these drug delivery systems to target infected macrophages in order to treat drug resistant TB (both MDR and XDR) [90]. All the studies mentioned in this section clearly depicted that bare/capped monometallic (AgNPs, AuNPs, citrate capped AuNPs, PAH capped AuNPs, ALG capped AgNPs, PVP capped AgNPs etc.) and bimetallic nanoparticles (Ag-AuNPs) can easily penetrate through the mycolic acid cell wall of *M. tuberculosis* and are the potential candidates for the treatment of tuberculosis (Figure 2 and Table 2).

**Table 2:** Potential usage of nanoparticles against tuberculosis

Class of nanoparticles	Various nanoparticles (NPs)	Brief description	Doses	Effect of NPs or mechanism of action	Nature of studies	References
Inorganic or Metallic	Citrate or polyallylamine hydrochloride stabilized gold nanoparticles (citrate or PAH AuNPs)	Prepared in an aqueous solution of HAuCl_4_ and citrate or PAH and in the presence of gold seed particles Average size of Citrate or PAH AuNPs: 20–30 nm	0.1, 1, 5 and 10 µg/ml of both citrate or polyallylamine hydrochloride stabilized gold nanoparticles	The growth of *E. coli* was more inhibited at lower concentrations of citrate capped AuNPs (0.1 and 1 μg/ml) and higher concentrations of PAH capped AuNPs (5 and 10 μg/ml)	*In vitro*	[86]
			0.1, 1, 5 and 10 µg/ml citrate AuNPs 1, 5, 10, 20 µg/ml PAH AuNPs	It was observed that the BCG fluorescence had decreased maximally upon the treatment with lower concentrations of citrate capped AuNPs (0.1 and 1 μg/ml) and higher concentrations of PAH-capped AuNPs (10 and 20 μg/ml)		
	Silver nanoparticles (AgNPs)	Synthesized by photo irradiation of AgNO_3_ in Triton X-100 solution for 60 min Average size of AgNPs: ∼30 nm	1 and 10 µg/ml	Effective antibacterial activities were observed in all concentrations		
			1, 5, 10 and 20 µg/ml	In all concentrations of AgNPs, similar fluorescence inhibition was observed		
	Silver nanoparticles (SNPs) stabilized by polyvinylpyrrolidone (SNPs-PVP)	Single dispersion of silver nanoparticles (SNPs) which was stabilized by PVP Average diameter of SNPs-PVP: 43.6 ± 10.7 nm	0.1, 1, 10, 25 and 50 µg/ml	Maximum inhibition of growth of *M. tuberculosis* H37Rv was achieved in presence of 50 µg/ml SNPs-PVP	*In vitro*	[85]
			0.1 mg/kg	After 10 days of exposure to SNPs-PVP, the mycobacterial load in lungs and spleen of mice had decreased drastically (two-fold). The inflammatory phenomenon in the mice lungs was decreased substantially due to the reduction of interleukin-4 level and the level of different immunological markers such as interferon-γ (IFN-γ), tumour necrosis factor-α (TNF-α) etc.	*In vivo* (Mouse model)	
	Alginate (ALG) capped silver nanoparticles (ALG-AgNPs)	An aqueous solution of AgNO_3_ was added dropwise to an aqueous solution of ALG (used as a stabilizing agent), glucose (used as a reducing agent), and NaOH at room temperature under stirring condition so as to synthesize ALG-AgNPs Average size of ALG-AgNPs: 69.7 ± 18.4 nm	25, 50 and 100 μg/ml	Inhibited the growth of MDR/XDR strains of *M. tuberculosis*. The growth of *M. tuberculosis* H37Rv was reduced appreciably in latency. Increased the cell permeability which may be the mechanism of anti-mycobacterial activity of ALG-AgNPs	Cell culture and *in vitro*	[81]
			200 μg/ml (Zebrafish model). 10, 50, 100 mg/kg (mouse model)	Reduced the bacterial load in the experimental animals (zebrafish and mouse) with no cytotoxicity to host cell	*In vivo* (zebrafish and mouse model)	
	Mesoporous silica nanoparticles containing supported silver bromide nanoparticles (MSNs-AgBrNPs); Ag@MSNs	One pot route synthesis method was used for the incorporation of AgBr into MSNs network. Hydrodynamic size of MSNs-AgBrNPs is 245.3 ± 4.8 nm. Ag@MSNs were prepared by chemical reduction of AgNO_3_ with sodium borohydride. Cetyltrimethylammonium bromide (CTAB) and Tetraethylorthosilicate (TEOS) were used as stabilizing and coating agent, respectively. Hydrodynamic size of Ag@MSNs is 91.3 ± 1.6 nm	Information not available	MSNs-AgBrNPs had shown efficient antimycobacterial effect in comparison to Ag@MSNs. The possible mechanism of better efficiency of MSNs-AgBrNPs over Ag@MSNs could be due to the distribution of AgBrNPs throughout the silica network and mesoporous channels; thus, more exposed to the media, in comparison to the Ag core present in Ag@MSNs. The morphological studies (carried out by cryo-EM) further revealed that MSNs-AgBrNPs exerted efficient anti-mycobacterial activity by damaging the cellular envelope of *M. tuberculosis* H37Rv	*In vitro*	[87]
	Biosynthesized AgNPs, AuNPs, and bimetallic Au-AgNPs	Biosynthesized from leaf extract of *B. prionitis* (A), root extract of P. zeylanica (B), and bark extract of *S. cumini* (C) Average size of AgNPs: 10–120 nm (A), 60 nm (B), 9–35 nm (C); average size of AuNPs: 15–35 nm (A), 20–30 nm (B), 1–60 nm (C); average size of Au-AgNPs: 10–70 nm (A), 90 nm (B), 10–20 nm (C)	Au-AgNPs dosage was 3 µg/ml	The bimetallic nanoparticles i.e. Au-AgNPs are much more efficient in context of anti-mycobacterial activities than the monometallic ones (AuNPs, AgNPs). Au-AgNPs synthesized from the bark extract of *S. cumini* plant, exhibited higher specificity and selectivity to the pathogen *M. tuberculosis*	*In vitro*	[88]
	Silver nanoparticles (AgNPs-PLGA)	Encapsulated to a biodegradable and biocompatible polymer (e.g. poly (D,L-lactide-co-glycolide); PLGA	Information not available	Effective antibiotics can be delivered directly to the infected area of alveolar macrophages	*In vitro*	[83]
	Gold nanorods (AuNRs) and gold nanospheres (AuNSs) conjugated with rifampicin (RF) (AuNRs@RF, AuNSs@RF)	AuNRs and AuNSs were prepared by using a seedless mediated growth technique and a citrate reduction of chloroauric acid (HAuCl_4_). These nanosystems were then conjugated with Rifampicin Approximate length and width of gold nanorods were 25 (± 3) × 5 (± 8) nm	5 and 10 nM	Cytotoxicity of AuNRs@RF was slightly more than that of AuNSs@RF. Both the nanosystems efficiently delivered rifampicin in the macrophage cells	Cell culture	[90]
	Silver nanoparticles conjugated with Vancomycin (AgNPs-VAM)	Prepared by citrate reduction of silver nitrate. With the help of n- hydroxysuccinimide and 1- ethyl-3-(3-diethylaminopropyl carbodiimide), vancomycin (VAM) was successfully loaded onto the surface of AgNPs. Approximate size of AgNPs-VAM: 30 ± 3 nm	Information not available	By conjugating with AgNPs, the uptake of VAM was significantly enhanced, resulting in improved delivery of the antibiotic into *Mycobacterium smegmatis*	*In vitro*	[91]
	Silver nanoparticles combined with different antibiotics	Aqueous solution of silver nanoparticles (2.5, 5, 25 and 50 mg/l) was combined with 1 mg of different antibiotics (isoniazid, rifampicin, streptomycin, kanamycin, ethambutol, ethionamide, levofloxacin, ofloxacin and cycloserine). Approximate size: 5–50 nm	2.5–5.0 mg/l (for *in vitro* studies) AgNPs (25 mg/kg) was combined with 50 mg/kg (for mouse model experiments)	The inhibition of growth of mycobacteria and mice survival rate were maximum with the usage of silver nanoparticles along with isoniazid	*In vitro* and *in vivo* (mouse model)	[92]

## Nanotechnology and leprosy

### Limitations of conventional leprosy therapy

Leprosy is a highly debilitating disease that has affected the mankind since the prehistoric times. Leprosy is treated by the Multidrug therapy (MDT) regimen introduced by the WHO in the 1980s. Despite the success of MDT in the treatment of leprosy, poor patient–drug compliance has been observed over the years due to the adverse side effects of these MDT drugs, namely rifampicin, dapsone and clofazimine [93]. These drugs, though highly effective against leprosy, are classified as Class II drugs according to the Biopharmaceutics Classification System as they have low water solubility and oral drug bioavailability [94]. Clofazimine tends to re-crystallize in cells and intestinal lumen due to the high dosage [95]. There are also incidences of antibiotic resistance cropping up due to such issues. Brazil, India and other parts of south-eastern Asia heavily contribute towards the global leprosy burden. Such problem demands the emergence of new therapeutic tools against leprosy. To counter the issues of poor patient–drug compliance and adverse side effects of these drugs towards patients, several research groups are working towards developing various nano-systems that would help in targeted delivery of these drugs. These nano-systems may potentially reduce the dosage of these drugs as well as promote sustained drug release on targeted site and also increases the bioavailability of these drugs. This approach also decreases the side effects associated with these drugs.

### Usage of nanoparticles/nanoformulations for the effective delivery of anti-leprotic drugs and treatment of leprosy

Dapsone (Dap) loaded pH sensitive nanoparticles were prepared by Chaves et al. by employing a copolymer Eudragit L100 (EL100), which is soluble at a pH above 6.0. Optimized using the Plackett Burman Design and Box-Behnken Design, the nanoparticles were of the size ∼198 nm. These nanoparticles retained the drug in simulated stomach environment at pH 1.2 and most of the drug was released upon reaching pH 6.8. This formulation was non-cytotoxic towards various cell lines (upto 400 µg/ml) and it also had a sustained drug release pattern with better permeability compared to free drug [96]. The same group also developed poly (D,L-lactide-co-glycolide); PLGA copolymer based clofazimine (Clz) nano-delivery system using Plackett Burman Design (particle size: ∼211 nm), which gave similar advantages over free clofazimine. These advantages could be attributed to the properties of copolymer PLGA. Degradation of PLGA matrix consequently helped sustained drug release [97]. They also used the combination of these two nano-systems (Clz-PLGA and Dap-EL100) and found efficient intestinal permeability. Such combination would also circumvent the free drug associated toxicity to an extent, avoiding intestinal damage, hence improving patient compliance [98]. Mesoporous silica nanoparticles loaded with high amounts of clofazimine by using acetophenone as a co-solvent were developed by Wei Chen et al. These nanoparticles released clofazimine in high amounts which enabled the killing of *M. tuberculosis* bacteria without affecting the macrophages [99]. Many solid-lipid nanoparticles (SLNs) have also been used for the effective delivery of different anti-leprotic drugs. Reis and coworkers utilized Box Behnken design for the optimization of mannosylated SLNs having particle size of ∼333.2 nm [100]. The release rate of drug from mannosylated SLNs coated dapsone depends on pH i.e., released faster at acidic pH as compared with neutral pH. This also targeted the intestinal microfold cells (M-cells) which transports antigens from intestinal lumen to the immune system and mannosylation helps in this uptake. These nanoparticles were stable for 8 weeks [100]. Another SLNs i.e., Lactonic sophorolipids stabilized with various non-ionic polymeric surfactants (Pluronic F68 and F127) were utilized by Kanwar et al., to prepare dapsone and rifampicin loaded SLNs whose drug release profile was modelled kinetically. It was observed that drug transport mechanism for dapsone and rifampicin are Fickian driven process and Non-Fickian process, respectively. It had high entrapment efficiency and loading capacity as well. Despite the high release rate of the drugs from the SLNs, the drug concentrations were well within therapeutic limits indicating controlled drug release [101,102].

Nanoemulsions derived from micellar solutions are efficient delivery systems which can increase drug absorption by protecting the drugs from hydrolysis and degradation [102–104]. Dapsone containing oral nanoemulsion and topical dapsone nanoemulsion was developed by Monteiro et al. [103] and Borges et al. [105], respectively. The dapsone oral nanoemulsion was developed using propylene glycol as a co-solvent and was a water in oil (w/o) system which was inverted *in vivo* to an oil in water (o/w) system. Such inversion increased the drug permeability as well as the drug dissolution compared with powdered dapsone [103]. Topical administration of drugs resulted in dose reduction that limited the adverse effects caused due to higher dosage of the drugs [105]. A topical dapsone nanoemulsion was developed by using isopropyl myristate and n-methyl pyrrolidone, where isopropyl myristate containing nanoemulsion showed higher *in vitro* epidermal permeation whereas n-methyl pyrrolidone had higher dapsone solubilization and *in vitro* release rate. These formulations were also stable for a period of 3 months [105]. The mode of action, anti-leprotic drug release along with other important details of all these above mentioned nanoparticles have been summarized in Table 3.

**Table 3:** Potential usage of nanoparticles/nanoformulations for the effective delivery of anti-leprotic drugs and treatment of leprosy

Class of nanoparticles	Various nanoparticles under each class	Brief description	Doses	Mode of action/drug release	Nature of studies	References
Polymeric nanoparticles	Dapsone loaded Eudragit L100 (copolymer) nanoparticles (Dap-EL100)	Optimized using Plackett Burman Design and Box-Behnken Design. Particle size: 198 ± 6 nm	No cytotoxicity upto 400 µg/ml in various cell lines	Sustained pH sensitive drug release at pH above 6	*In vitro*	[96]
	PLGA copolymer based Clofazimine nanoparticles (Clz-PLGA)	Optimized using Plackett Burman Design. Particle Size: 211 ± 3 nm	Information not available	Sustained drug release when PLGA matrix is degraded	*In vitro*	[97,98]
Solid Lipid Nanoparticles	Mannosylated Solid Lipid nanoparticles coated dapsone	Optimized using Box-Behnken Design. Particle size: 333.2 ± 2.3 nm	Information not available	pH sensitive drug release (faster release at acidic pH)	*In vitro*	[100]
	Lactonic sophorolipids + polymeric surfactant (Pluronic F68, F127) + dapsone/rifampicin	Prepared using solvent injection method. Particle size: F68: 144 nm radii F127: 136 nm radii	Information not available	Drug transport mechanism for dapsone is Fickian driven process, for rifampicin it is Non-Fickian process	*In vitro*	[101,102]
Nanoemulsions	Nanoemulsion containing dapsone	Prepared using propylene glycol as co-solvent	Information not available	Water in oil system inverted *in vivo* to an oil in water system leading to drug release	*In vitro*	[103]
	Topical dapsone nanoemulsion	Isopropyl myristate/n-methyl pyrrolidone based dapsone nanoemulsion	Information not available	isopropyl myristate containing nanoemulsion showed higher *in vitro* epidermal permeation, n-methyl pyrrolidone had higher dapsone solubilization and *in vitro* release rate	*In vitro*	[105]
Inorganic	Clofazimine loaded mesoporous silica nanoparticle	Acetophenone was used as a co-solvent for loading clofazimine into the mesoporous silica nanoparticles	0.5, 1 and 2.0 µg/ml of Clofazimine	Acetophenone assisted clofazimine drug delivery	*In vitro*	[99]
	Nanogold and silver nanoparticles	Nanogold were synthesized by the reduction of HAuCl_4_ with sodium citrate. Silver nanoparticles were produced by the chemical reduction of silver nitrate	Information not available	Combination of nanogold-AgNPs was effective in reducing the wound width by 80% in patients under the MDT regimen; while the wound width of the control group had decreased only by 50%. But mechanism is unknown	*In vivo*	[106]
	Gold and Silver nanoparticles	Commercially purchased citrate capped AuNPs and AgNPs. Particle Size: ∼20 nm	0.1–1.5 μM of AuNPs/AgNPs (Chaperone assays) and 0.6 μM of AuNPs/AgNPs (other assays)	HSP18–AgNPs interaction causes oligomeric dissociation, decreased surface hydrophobicity and reduced chaperone activity of HSP18. Opposite effects were observed in HSP18 due to AuNPs interaction	*In vitro*	[108]

**Figure 3:**
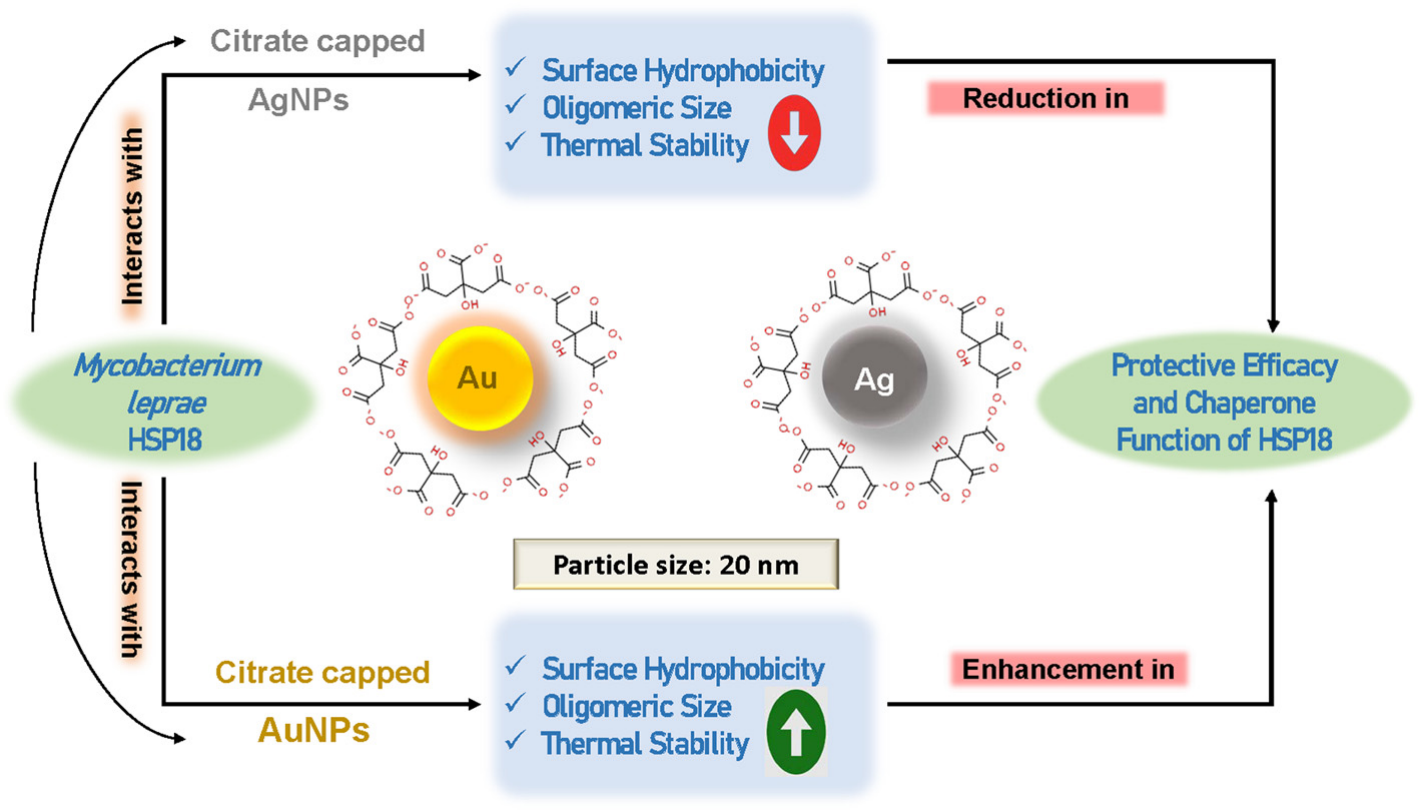
Effect of citrate capped gold and silver nanoparticles (AuNPs and AgNPs) on the structure and chaperone function of *M. leprae* HSP18 Schematic representation of the differential effect of these two nanoparticles on the structure and chaperone function of HSP18 (which plays an important role in the growth and survival of *M. leprae* pathogen inside hosts).

Meanwhile, Taufikuromah et al. used a combination of gold and silver nanoparticles to topically treat epidermal wounds due to leprosy. They found that such a combination was effective in reducing the wound width by 80% in patients under the MDT regimen while the wound width of the control group had decreased only by 50% (Table 3) [106]. However, the mode of action of these two nanoparticles towards the inhibition of growth and survival of *Mycobacterium leprae* (leprosy causing pathogen) is mostly unclear. The non-culturable nature of this pathogen in artificial medium hinted towards this understanding. Our group has taken an indirect approach to understand the possible mode of action of these two nanoparticles. The effect of ∼20 nm citrate capped silver nanoparticles (AgNPs) and gold nanoparticles (AuNPs) on HSP18, an 18 kDa immunodominant small heat shock protein belonging to *M. leprae* was tested. This small heat shock protein is also responsible for the growth and survival of this pathogen in host macrophages by virtue of its chaperone function [93,107]. Fluorometric characterization of binding of these two nanoparticles with HSP18 revealed the nature/stoichiometry of binding i.e., one silver nanoparticle/gold nanoparticle binding per subunit of HSP18 with binding affinity in the submicromolar range [108]. It was further found that the interaction of these two nanoparticles with HSP18 affects the structure, chaperone function and protective efficacy of HSP18 in a distinctly opposite manner. HSP18–AgNPs interaction led to oligomeric dissociation, decreased surface hydrophobicity, thermal stability, protective efficacy and reduced chaperone function of HSP18, while all of these were enhanced upon interaction of HSP18 with AuNPs (Figure 3 and Table 3) [108]. This particular study evokes the possibility of nanoparticles for the effective treatment of leprosy by targeting different overexpressed antigenic proteins within the pathogen during infection [108]. In future, nanoformulations that have the ability to target HSP18, an important mycobacterial small heat shock protein, could be employed for treating leprosy patients. It is noteworthy to mention here that heat shock proteins especially large HSPs are the important drug targets in cancer and nanotechnology has been extensively used to carry their inhibitors to the cancer cells.

## Nanotechnology-based approaches for the treatment of cancer by targeting large heat shock proteins

### HSP90: an important drug target in cancer

Cancer is a generic term for a group of diseases that possess serious threat to public health globally. It is characterized by the development of abnormal cells formed by the uncontrolled cell division which eventually leads to tissue destruction. Considering its diverse forms, high lethality and widespread occurrence in population, designing of specific diagnosis as well as effective treatment is a huge challenge to combat this disease. Currently, various approaches have been implemented for the specific diagnosis and therapeutic development for cancer. However, conventional diagnostic and therapeutic approaches for cancer are severely threatened by the typical features of malignant tumours, including their infinite capability for replication, metastasizing ability, immune evasion and heterogeneity [109,110]. Presently, numerous genes, active enzymes and proteins in connection to different stages of cancer manifestation are being targeted for the proper diagnosis and effective treatment of cancer [111,112]. One such target is a large heat shock protein namely HSP90 which has been widely investigated and different HSP90 targeting molecules have reached clinical trials.

HSP90 plays an essential role in many physiological activities [113,114]. HSP90 family proteins are a group of evolutionarily conserved protein found in eukaryotic cells, having monomeric molecular weight of ∼90 kDa. HSP90 acts as molecular chaperones by aiding in the proper folding of newly synthesized polypeptide chains and preventing the aggregation of stressed client proteins [113,114]. Members of mammalian HSP90 family are located in various cellular compartments- HSP90 α and HSP90 β in the cytoplasm, glucose-regulated protein 94 (GRP94) in the endoplasmic reticulum (ER) and tumour necrosis factor receptor-associated protein 1 (TRAP1) in the mitochondrial matrix [113,115]. These proteins are involved in important cellular processes and biochemical pathways like apoptosis, cell cycle control, cell survival, protein folding and degradation and cell signalling [113–115]. It is well known that HSP90 plays a vital role in protein homeostasis, cell differentiation and development [113–115]. However, overexpression of HSP90 is related to many diseases, including viral infections, inflammation, neurodegenerative diseases and several types of cancers [113–115].

It has been reported that HSP90 performs critical roles in maintaining the activity, proper folding, stability, functioning and proteolysis of several oncoproteins in various types of cancers [116]. It is usually overexpressed in cancer cells which lead to increase in tumour growth, adhesion, invasion, metastasis and angiogenesis [117]. Moreover, it has been shown that cancer cell epithelial–mesenchymal transition (EMT), invasion, migration and tumour metastasis are all facilitated by HSP90 expression, in accordance with the activation of hypoxia-inducible factor 1α (HIF-1α) and nuclear factor kB (NF-kB) [115]. Furthermore, HSP90 was shown to regulate the transcription and expression of vascular endothelial growth factor receptor (VEGFR), which is a key molecule involved in the process of angiogenesis. Hence, the overexpression of HSP90 could potentially increase tumorigenesis, migration, penetration and angiogenesis [118]. It is also known that the expression level of HSP90 can serve as a possible biomarker for a number of different cancers including, lung cancer, esophageal carcinoma, bladder cancer, melanoma, leukemia, non-small cell lung cancer (NSCLC) and breast cancer [119–122].

### Different HSP90 inhibitors and their transportation in different cancer cells using nanosystems

As HSP90 is an important drug target, investigators have developed several HSP90 inhibitors. These inhibitors are broadly classified into two categories: (i) N-terminal HSP90 inhibitors; examples are geldanamycin (GA), tanespimycin or 17-allylaminogeldanamycin (17-AAG), radicicol and 17-(2-dimethylaminoethyl)amino-17-demethoxygeldanamycin (17-DMAG) [123–126]. (ii) C-terminal HSP90 inhibitors such as novobiocin (nvb), 6-bromo-3-[4-methoxyphenylcarboxamide]-quinolein-2-one (BrCaQ), cyclic peptide analogs (SM122, SM145 and SM253) [127,128]. The most commonly used FDA approved HSP90 inhibitor is 17-AAG. Despite its selective mechanism of action on cancer cells, 17-AAG faces challenging issues due to its poor aqueous solubility, short biological half-life and hepatotoxicity which hindered its progression into clinical trials. Nanotechnology based approach has been employed to overcome these problems and to deliver 17-AAG and other HSP90 inhibitors in different cancer cells. Xioang et al. have reported the use of degradable amphiphilic diblock polymers of poly(ethylene oxide)-block-poly(D,L-lactide) [PEO-b-PDLLA] as nano-carriers which increased the aqueous solubility of 17-AAG by 150-fold as compared with the solubility of 17-AAG in absence of these nano-carriers [129]. Use of nano-carriers has increased the half-life of 17-AAG in serum and blood along with an increased volume of distribution in rat model [129]. Chandran et al., have formulated PEG-DSPE micellar nano-carriers (prepared using dry film method) for the delivery of 17-AAG without the inclusion of any organic solvent [130]. They have found that by modulating the PEG-DSPE concentration and incorporation of D-α-tocopheryl polyethylene glycol 1000 succinate (TPGS) in the micelle composition, has decreased the release of 17-AAG significantly from the micelles [130]. In an independent study, Hussain and co-workers synthesized polylactide-co-glycolide-polyethylene glycol-folic acid (PLGA-PEG-FA) using nanoprecepitation method. Then, this nanoparticles containing 17-AAG were characterized and studied their cellular uptake and cytotoxicity in MCF-7 breast cancer cell lines. They observed a superior cellular uptake and increased cytotoxicity of folate targeted nanoparticles in comparison to free 17-AAG [131]. The same group also synthesized Pluronic® P-123 and F-127 mixed micelles loaded with 17-AAG using thin film hydration method which can internalize inside the human brain tumour glioblastoma cell lines [132]. These 17-AAG loaded mixed micelles showed increased cytotoxicity to the glioblastoma cells as compared with free drug. Thus, this particular study hinted towards usage of 17-AAG loaded Pluronic® P-123 and F-127 mixed micelles for the treatment of the most common and aggressive malignant primary brain tumour in human glioblastoma multiforme (GBM) [132]. Polymeric micelles carrying 17-AAG were synthesized by Larson et al., using poly(styrene-*co*-maleic acid) [SMA] copolymers that exhibited potent *in vitro* activity against DU145 human prostate cancer cells [133]. This nanoformulation was also well tolerated and exhibited potent anti-cancer activity in mouse model harbouring prostate cancer tumour xenograft. Von Hoff and co-workers had commenced a Phase I trial of a nanoemulsion of 17-AAG, namely CNF1010, but due to the toxicity issue, it was dropped from clinical trials [134]. In another Phase II clinical trial carried out by Tao et al., the drug 17-AAG was successfully converted to albumin-bound nanoparticles (nab-17-AAG) but the trails were withdrawn later [135]. Similarly, a study carried out by Won et al. described a self-assembled biodegradable recombinant human gelatin (rHG) modified with α-tocopheryl succinate (TOS) nanoparticles via EDC/NHS reaction that was efficiently encapsulated with 17-AAG [136]. This 17-AAG-loaded nanoparticles were non-immunogenic and more efficient than free 17-AAG. Along with this, from studies executed in tumour mouse model, the enhanced permeability and retention effect of nanoconjugated 17-AAG was evident [136]. Rubinstein and co-workers used co-precipitation/reconstitution method to solubilize 17-AAG into extendable (PEGylated), biocompatible and biodegradable sterically stabilized phospholipid nano-micelles that increased intracellular uptake of 17-AAG-loaded phospholipid nanomicelles thus, amplifying the drug potency and its cytotoxicity to MCF-7 human breast cancer cells [137].

Additionally, co-administration of polymer-based nanoshells which consists of multiple drugs along with 17-AAG was initiated. Kwon and his co-workers designed a 3-in-1 polymeric micelle nano-container for delivering three drugs that includes, 17-AAG (HSP90 inhibitor), rapamycin and paclitaxel [138]. The polymeric nano-carrier poly(ethylene glycol)-block-poly(d,l-lactic acid) [PEG-b-PLA] micelles were capable to solubilize the drugs. According to the findings of this study, micelles composed of 3-in-1 PEG-b-PLA exerted a powerful synergistic effect in breast cancer cell lines (MCF-7 and 4T1) [138]. Notably, intravenous (IV) injection of 17-AAG, paclitaxel and rapamycin using micelles composed of 3-in-1 PEG-b-PLA was well tolerated by FVB albino mice [138]. In an independent study, Kozak and co-workers formulated a multidrug-loaded nano-micelle, triolimus, containing paclitaxel, rapamycin and 17-AAG [139]. The combination of these three drugs in nano-micelle exhibited potent cytotoxic synergy against lung cancer cell line (A549) and breast cancer cell line (MDA-MB-231). This nano-drug conjugate had an inhibitory effect on the Ras/Raf/mitogen-activated protein kinase pathway as well as the PI3K/Akt/mTOR pathway [139]. In a tumour xenograft mouse model, using this nano-micelle triolimus, a delay in tumour growth as well as a 50% reduction in tumour cell proliferation was observed as compared to paclitaxel-containing micelles. Similar kind of study was performed using different combinations of drugs in nano-carrier were found to be effective against a wide range of cancers. Some of the examples are: 17-AAG/Paclitaxel combination in polyethylene glycol-poly(D, L-lactic acid) [PEG-PLA] and PEG-distearoylphosphatidylethanolamine/tocopheryl polyethylene glycol 1000 (PEG-DSPE/TPGS) nano-micelles against human ovarian cancer [140], 17-AAG/Docetaxel drugs in hyaluronic acid (HA)-decorated poly(lactic-co-glycolic acid) (PLGA) nanoparticles [HA-PLGA NPs] against squamous cell carcinoma [141], 17-AAG/Doxorubicin combination in polypeptide based nanogels against ErbB2-driven breast cancer [142].

Beside 17-AAG, another N-terminal HSP90 inhibitor is geldanamycin (GA). Attempt has been taken for delivery of this anti HSP90 inhibitor utilizing nanoformulations. Forrest and coworkers have formulated a lipophilic GA prodrug, 17'GAC(16)Br encapsulated in acid-catalyzed ring opening polymerization mediated methoxy-capped poly(ethylene glycol)-block-poly(epsilon-caprolactone) [mPEG-b-PCL] micelles [143]. This micellar nanoformulation exhibited significant accumulation and lower systemic toxicity in rat models. Semi-synthetic derivative of geldanamycin and 17-DMAG (another important N-terminal HSP90 inhibitor) were also delivered into cancer cells by utilizing nano-carriers. It has been reported that mRNA expression of HSP90 gene in lung cancer and breast cancer cell lines was inhibited by 17-DMAG, which was physically encapsulated in PLGA-PEG (poly (DL-lactic-co-glycolic acid)-polyethyleneglycol) nanoparticles. PLGA-PEG was synthesized using double emulsion method [144,145]. This nanoencapsulated complex exhibited higher inhibitory effect on lung cancer A549 cell line and T47D breast cancer cell line as compared to 17-DMAG alone [144,145]. SNX-2112 is another N-terminal HSP90 inhibitor and a promising anticancer agent but has poor solubility. To enhance its solubility, Wu and co-workers developed a nanocrystal formulation for SNX-2112 and found that, due to their cosolvent-like pharmacokinetic behaviour, the nanocrystals rapidly released the SNX-2112 drug molecules in *in vivo* rat-model [146]. The same group further formulated glucose-based mesoporous carbon nanospheres (MCNs) using hydrothermal reaction to efficiently load SNX-2112 which significantly enhanced the cellular uptake, biodistribution and anti-tumour effect in xenograft of breast cancer of mice model [147].

Scientists have also utilized various nano-carrier to deliver different C-terminal HSP90 inhibitors into cancer cells. Sansalvamide A derivatives, a novel series of C-terminal HSP90 inhibitors were synthesized by Kim et al. and conjugated them to the star polymer core to generate nanoparticles [148]. These polymer conjugated HSP90 inhibitors induced apoptosis by a caspase 3-dependent pathway and also imparted cytotoxicity to a colon cancer cell line [148]. Sauvage et al. have loaded another C-terminal HSP90 inhibitor, 6BrCaQ [N-(6-bromo-1-methyl-2-oxoquinolin-3-yl)-4-methoxybenzamide] into nanometre-scaled liposomes and examined their suitability for drug delivery to solid tumours. Administration of liposomal 6BrCaQ showed decrease in HSP90 expression and significant anticancer activity towards prostate cancer cell lines [149].

Interestingly, one novel gene therapy strategy was taken to deliver micro-RNA (miRNA) of HSP90 using nanotechnology approach. Banerjee and co-workers designed an artificial anti-HSP90 miRNA plasmid vector which was delivered through a glucocorticoid receptor (GR) targeted liposome complex for targeting and down-regulation of HSP90 in cancer cells [150]. GR-mediated delivery of miRNA-HSP90 plasmid in tumour-bearing mice enforced apoptosis in angiogenic vessels and in tumour mass as well as significantly shrunk tumour-volume [150]. As a whole, this unique strategy showed significantly higher anti-cancer activity against tumour models of melanoma and lung cancer.

### Utilization of inhibitors of HSP90 and HSP70 in cancer thermotherapy

The delivery of these anti-HSP90 nanoformulations also has usage in the thermotherapy of cancer. Thermotherapy involves heating the tumour to cause local hyperthermia. One of the major concerns impeding the advancement of this therapy is the damage of adjacent healthy tissues due to heating. Hyperthermia also causes a rise in the expression of heat shock proteins, notably HSP90 and HSP70, which counteracts the induction of apoptosis and makes the cell resistant to thermal injury. To overcome this limitation, nanotechnology-based approaches have been adopted for precise and selective ‘intracellular’ heating along with diminishing the protective functions of HSP90 and/or HSP70. For example, Fraifeld and co-workers have described that human ovarian cancer cells (SKOV-3) exhibited a greater sensitivity to hyperthermia when treated with geldanamycin (GA) and novobiocin (nvb) [151]. Furgeson’s group reported that poly(K)_8_-poly(VPGXG)60 block copolymers [K_8_-ELP(1-60)] conjugated with geldanamycin can be used in conjunction with heat to produce an effective chemotherapy regimen [152]. In addition to these, HSP90 inhibitors in conjugation with various nano-encapsulation were co-delivered with photothermal agents to reduce the thermo-resistance of cancer cells [153–156].

Attempts have also been made to produce hyperthermia utilizing nanoformulations containing HSP90 inhibitor. Liposomes loaded with HSP90 inhibitor and iron oxide nanoparticles (also known as magnetoliposomes) generated heat when exposed to a magnetic field. This form of liposome was employed to encapsulate 17-AAG in a recent study, and it was coated with an antibody targeting CD90 to improve uptake by tumour stem cells [157]. Furthermore, *in vitro* and *in vivo* experiments using liver cancer stem like cells (LCSCs) were carried out so as to assess hyperthermia inducing potency of this magnetic nanoformulation. Over-expression of HSP90 was predicted to be observed in LCSCs after exposure to magnetic hyperthermia. Magnetoliposomes targeted to CD90 that included 17-AAG showed greater anti-tumour effects upon thermotherapy compared to magnetoliposomes without the encapsulated drug. Recently, the effectiveness of 17-AAG in poly (lactic-co-glycolic acid) nanoparticles loaded with Fe_3_O_4_ for the treatment of pancreatic cancer was evaluated by Rochani et al. [158]. The pancreas cancer cell line has been shown to lose 75% of its viability after being subjected to magnetic field induced hyperthermia for 3 h. This finding demonstrates the effectiveness of combining heat and heat shock protein suppression in cancer treatment [158].

**Figure 4:**
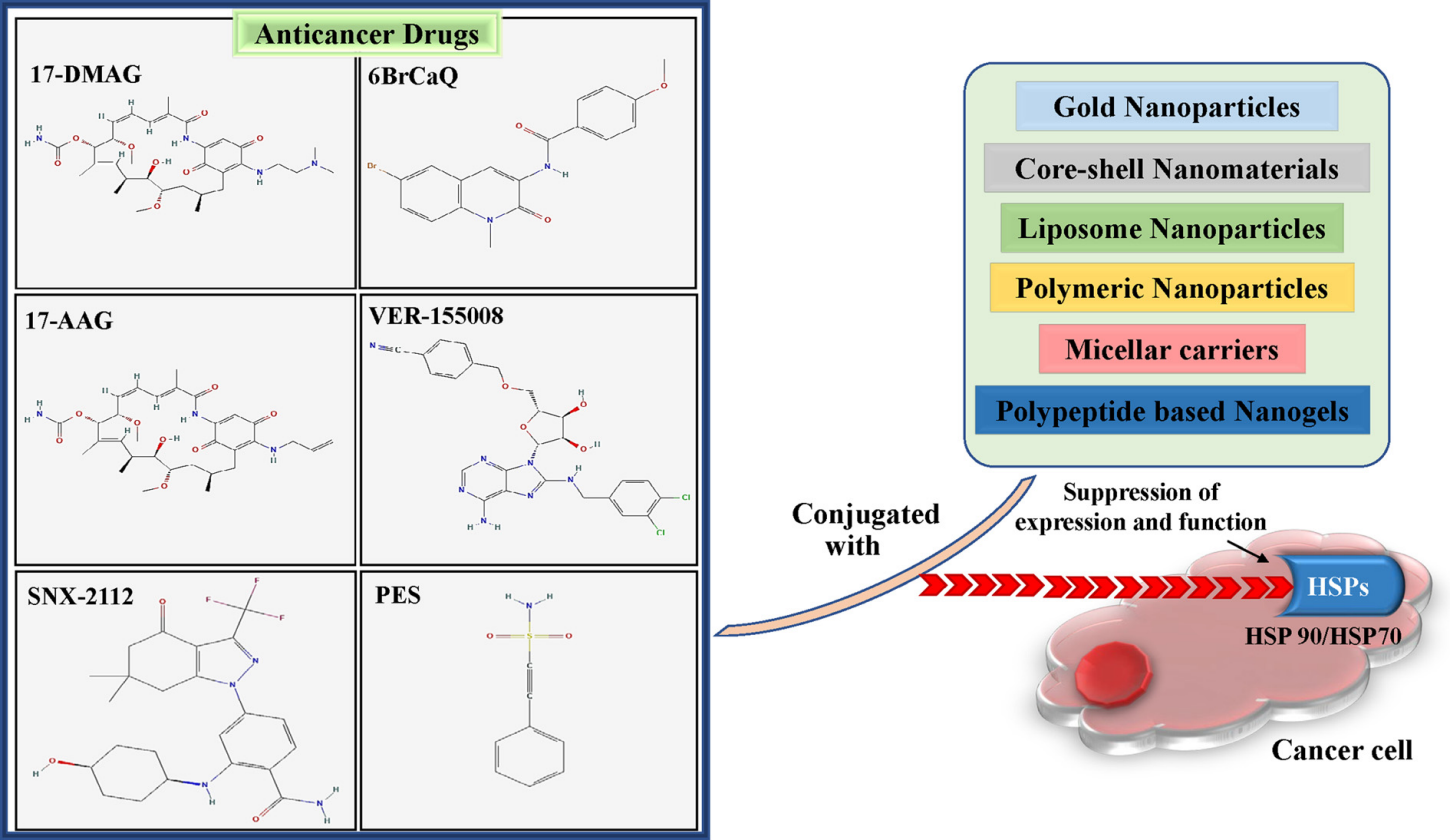
Application of nanotechnology in cancer Schematic depiction of the potential of HSP90 inhibitor/HSP70 inhibitor based nanoformulations towards the treatment of different types of cancer; 17-AAG, 17-allylaminogeldanamycin; 6BrCaQ, N-(6-bromo-1-methyl-2-oxoquinolin-3-yl)-4-methoxybenzamide; 17-DMAG, 17-(2-dimethylaminoethyl)amino-17-demethoxygeldanamycin; PES, 2-phenylethynesulfonamide; SNX-2112, 4-[6,6-dimethyl-4-oxo-3-(trifluoromethyl)-5,7-dihydroindazol-1-yl]-2-[(4-hydroxycyclohexyl)amino]benzamide; VER-155008, 5′-O-[(4-Cyanophenyl)methyl]-8-[[(3,4-dichlorophenyl)methyl]amino]-adenosine.

Apart from HSP90, another important large heat shock protein which has been targeted in nanoparticles based thermotherapy of cancer is HSP70. Gold nanoparticles (AuNPs) are used as drug (some well-known HSP70 inhibitors) carriers to treat various types of cancer. These nanoparticles, due to their EPR effect, can easily accumulate in tumours. More importantly, the ability of gold nanoparticles to convert light energy into heat, makes them important candidates for tumour-selective photothermal therapy. To find out the effect of VER-155008, a well known HSP70 inhibitor, on the sensitivity of tumour cells to heat, Tang et al. conjugated VER-155008 with methoxy-polyethylene-glycol-coated-gold-nanorods (MPEG-AuNR) [159]. It has been found that VER-155008 micelles down-regulated the expression of HSP70, thus attenuated the heat-resistance of tumour cells and enhanced the therapeutic outcome of mild-temperature photothermal therapy against colon cancer [159]. In an independent study, where Wang and co-workers employed core–shell structure Au@SiO_2_ nanomaterials with high photothermal performance conjugated with an HSP70 inhibitor 2-phenylethynesulfonamide (PES) into breast cancer tumour cells and exposed them to near-infrared light (NIR), they found that this irradiation generated mild heat induced apoptosis or necrosis at relatively low temperature [160]. All evidences presented in this section clearly depicted the immense potential of HSP90 inhibitor/HSP70 inhibitor based nanoformulations towards the treatment of different types of cancer (Figure 4 and Table 4).

**Table 4:** Potential usage of various nanoparticles against cancer by targeting large heat shock proteins

Target	Drug	Nanoparticles carrier	Brief description	Cancer type	Nature of studies	Reference
HSP90	17-AAG	poly(ethylene oxide)-block-poly(D,L-lactide) [PEO-b-PDLLA]	17-AAG and PEO-b-PDLLA (PEO and PDLLA) was dissolved in dimethylacetamide.	Prostate and breast cancer	*In vitro* and *in vivo*	[129]
		1,2-distearoyl-sn-glycero-3- phosphoethanolamine-N- [methoxy(polyethylene glycol)-2000]/d-α-tocopheryl polyethylene glycol 1000 [PEG-DSPE/TPGS]	Prepared using dry film method and PEG-DSPE, TPGS and 17-AAG were dissolved in chloroform.	Ovarian cancer	*In vitro* and *in vivo*	[130]
		polylactide-co-glycolide- polyethylene glycol-folic acid (PLGA-PEG-FA) nanoparticles	Nanoprecipitation method was used. 17-AAG and polymer were dissolved in acetone.	Breast cancer	*In vitro* and *in vivo*	[131]
		Pluronic® P-123 and F-127 mixed micelles	Prepared using thin film hydration method and was dissolved in ethanol along with 17-AAG.	Human brain tumour glioblastoma	*In vitro* and *in vivo*	[132]
		poly(styrene-*co*-maleic acid) [SMA] copolymer micelles	Tanespimycin (dissolved in minimal DMSO) was added to SMA solution dropwise.	Human prostate cancer	Phase I clinical trial (withdrawn)	[133]
		human gelatin (rHG) modified with alpha-tocopheryl succinate (TOS) nanoparticles	rHG-TOS was formed by EDC/NHS reaction. DMSO was used to dissolve it and 17-AAG.	General	*In vivo* and *ex vivo*	[136]
		PEGylated nano-micelles	Formed using co-precipitation/reconstitution method. DSPE-PEG_2000_ and 17-AAG were dissolved in methanol.	Human breast cancer	*In vivo*	[137]
		3-in-1 PEG-b-PLA micelles	PEG-b-PLA, PTX, 17-AAG and RAP were dissolved in acetonitrile.	Breast cancer	*In vivo* and *ex vivo*	[138]
		multidrug-loaded nano-micelles	PEG-PLA, paclitaxel, 17-AAG and rapamycin were dissolved in acetonitrile.	Lung and breast cancer	*In vivo* and *ex vivo*	[139]
		polyethylene glycol-poly(D, L-lactic acid) [PEG-PLA] nano-micelles	Prepared using solvent evaporation method. Paclitaxel, 17-AAG and co-polymer were dissolved in chloroform.	Human ovarian cancer	*In vivo*	[140]
		PEG-distearoylphosphatidyle thanolamine/tocopheryl polyethylene glycol 1000 [PEG-DSPE/TPGS] nano-micelles	Prepared using solvent evaporation method. Paclitaxel, 17-AAG and co-polymer were dissolved in chloroform.	Human ovarian cancer	*In vivo*	[140]
		hyaluronic acid (HA)-decorated poly (lactic-co-glycolic acid) (PLGA) nanoparticles [HA-PLGA NPs]	Prepared by o/w emulsification and solvent evaporation technique. PLGA, DTX and 17-AAG were dissolved in ethyl acetate.	Squamous cell carcinoma	*In vivo* and *ex vivo*	[141]
		polypeptide based nanogels	Nanogels were prepared using PEG-b-PPGA/Ca^2+^ block ionomer complex.	ErbB2-driven breast cancer	*In vivo* and *ex vivo*	[142]
		magnetoliposomes	Synthesized using co-precipitation method. Encapsulation of drug in PLGA was preformed using solvent evaporation and self-assembly technique.	Liver cancer stem like cells (LCSCs), pancreatic cancer	*In vitro* and *in vivo*	[157,158]
	Geldanamycin	methoxy-capped poly(ethylene glycol)-block-poly(epsilon- caprolactone) [mPEG-b-PCL] micelles	Synthesized by acid-catalyzed ring opening polymerization and was dissolved in acetone with prodrug.	Colon cancer	*In vivo* and *ex vivo*	[143]
		ELP K_8_-poly(VPGXG)60	Prepared using recursive directional ligation method and its conjugates were dissolved in DMSO.	Ovarian cancer	*In vivo*	[152]
	17-DMAG	poly (D,L-lactic-co-glycolic acid)-polyethyleneglycol [PLGA-PEG]	Synthesized using double emulsion method and encapsulated physically with drug. Dissolved in chloroform.	Lung cancer and breast cancer	*In vivo*	[144,145]
	SNX-2112	glucose-based mesoporous carbon nanospheres (MCNs)	Prepared using hydrothermal reaction and glucose was used as carbon source.	Breast cancer	*In vivo*, *ex vivo*	[146,147]
	Sansalvamide A derivatives	star polymer	Synthesized using reversible addition-fragmentation chain transfer polymerization technique.	Colon cancer	*In vivo*	[148]
	6BrCaQ	liposomes	Prepared using thin film hydration method. Cholesterol, phospholipids and 6BrCaQ were dissolved in chloroform.	Prostate cancer	*In vivo*	[149]
	miRNA	GR-mediated liposomes	DO liposome was formed by drying chloroform solution of DODEAC & cholesterol and DX liposome was prepared by drying chloroform solution of DODEAC, cholesterol & Dex.	Melanoma and lung cancer	*In vivo* and *ex vivo*	[150]
HSP70	VER-155008	methoxy-polyethylene-glycol- coated-gold-nanorods (MPEG-AuNR)	AuNR formed by seed mediated method and MPEG-thiol was added to AuNR solution to form MPEG-AuNR.	Colon cancer	*In vivo* and *ex vivo*	[159]
	PES	core-shell structure Au@SiO_2_ nanomaterials	Gold rods were coated with mesoporous silica to form Au@SiO_2_ nanocomposites.	Breast cancer tumour cells	*In vitro* and *in vivo*	[160]

## Toxicity of nanoparticles

Irrespective of the several added advantages of nanoparticles over traditional therapeutic agents, the success rate of nanoparticles to be utilized clinically is majorly halted due to their toxicity. Nanotoxicity is of great concern particularly in case of nanoparticles mediated drug delivery process. It has been reported that cationic nanoparticles impart more toxicity compared to that of anionic nanoparticles [1]. Interestingly, some of the inherent characteristics of nanoparticles like higher surface reactivity, higher surface-to-volume ratio etc., contribute towards their toxicity [161]. When nanoparticles interact with different biomolecules especially with protein, ‘protein-corona’ forms, such formation sometimes alters the pharmacological behaviour of nanoparticles in such a manner which could induce toxicity [162]. Basically, variable interaction or affinity of different nanoparticles with this protein corona can generate reactive oxygen species (ROS) which consequently causes cellular damage. Metal based nanoparticles can also produce ROS through surface modulation and through Fenton, Fenton-like and Haber-Weiss reactions [163]. Several attempts have been made to assess the toxic potential of nanoparticles of different blood cell components [164–170]. Huang and co-workers revealed the toxic effect of silver nanoparticles to red blood cells (RBCs) [164]. This group convincingly showed that the silver nanoparticles of 15 nm diameter induce more haemolysis and membrane damage than silver nanoparticles having larger size (50 and 100 nm). Some other inorganic nanoparticles (TiO_2_ and ZnO nanoparticles) induced toxicity also and increase haemolysis as well as the monocyte-derived dendritic cell (MDDC) death [165]. The size of nanoparticles also contributes towards their toxicity. Battal et al. demonstrated that the size of SiO_2_ nanoparticles greatly influence its genotoxic/mutagenic and cytotoxic effects [166]. These two effects of nanoparticles also depend on surface coating. Magdolenova et al. demonstrated that iron oxide coated with oleate are more cytotoxic and genotoxic than uncoated iron oxide nanoparticles [167]. When Delogu and co-workers subjected various pre-coated nanocapsules (Pluronic coated, Chitosan coated and Polyethylene glycol coated) to human primary immune cells, they observed that these nanocapsules differentially affect immune cells [168]. Yu and co-workers studied the effect of multiwall carbon nanotube on the DNA damage and cytotoxicity in male human peripheral blood lymphocytes and they found that these nanoparticles were able to induce considerable DNA damage and cytotoxicity [169]. In a separate study, Sun and co-workers reported that silicon nanoparticles can induce cytotoxicity and inflammation in human umbilical vein endothelial cells (HUVECs) by activating potassium channels [170]. Since, nanoparticles can induce toxicity when they come in contact with our blood cells, optimization of the biocompatibility of the nanoparticles must be looked upon very carefully. More importantly, the degradation process of nanoparticles inside body should be investigated carefully to overcome the toxic effects of nanoparticles.

## Conclusions and promising future prospective

The introduction of nanotechnology and nanomaterials had led to revolutionary changes in various domains of medical science. This review has presented an overview of the current advances in nanotechnology for the treatment of four important diseases (Alzheimer’s disease, tuberculosis, leprosy and cancer). The studies presented here clearly suggest that the ability of penetrating the blood–brain barrier makes nanoparticles excellent candidates to be applied in AD treatment, especially by inhibiting the fibrillation process and toxic effects of amyloid beta peptide. However, in future, a systematic study in this research field by exploring more efficient biodegradable nanomaterials that can be tagged with several disease modifying agents to successfully treat this disorder is highly demanding. Furthermore, it can be concluded that metallic nanoparticles are more useful towards the treatment of tuberculosis and leprosy possibly due to their ability to enhance the permeability of mycolic acid cell walls. Since, multimetallic nanoparticles exert better antimycobacterial activity than the monometallic nanoparticles, attempts should be made to screen the antimycobacterial potency of various other multimetallic nanoparticles. This futuristic approach could provide promising candidate(s) for the treatment of these two infectious diseases. This report also concludes that inorganic and/or organic based nanoparticles can be efficiently utilized for the delivery of various drugs used in tuberculosis, leprosy and cancer. Additionally, considering the toxic effects of nanoparticles, the evaluation of their biodegradability and cytotoxicity is extremely required. Such evaluation will certainly help us to assess their safe usage in drug delivery. Lastly, in future, more emphasis should be given to prepare non-toxic and biocompatible nanoparticles with improved pharmacokinetic behaviour, for their safer biomedical applications.

## Abbreviations

AgNP-VAM: silver nanoparticles with vancomycin
ALG-AgNPs: alginate-capped silver nanoparticles
APP: amyloid precursor protein
Aβ: amyloid β
BBB: blood–brain barrier
BPL: broncho-pulmonary lavage fluid
DTDTPA: dithiolated diethylenetriaminepentaacetic acid
EGFR: epidermal growth factor receptor
HUVEC: human umbilical vein endothelial cell
MDDC: monocyte-derived dendritic cells
MDR-TB: multidrug-resistant tuberculosis
NP: nanoparticle
PLGA: poly (D,L-lactide-coglycolide)
RR-TB: rifampicin-resistant tuberculosis
SLN: solid lipid nanoparticles
SNP- PVP: silver nanoparticles stabilized with polyvinylpyrrolidone
SPIO: superparamagnetic iron oxide
XDR TB: extensively drug-resistant tuberculosis

## References

[1] Chenthamara D., Subramaniam S., Ramakrishnan S.G., Krishnaswamy S., Essa M.M., Lin F.-H. et al. (2019) Therapeutic efficacy of nanoparticles and routes of administration. Biomaterials Res. 23, 20 10.1186/s40824-019-0166-x PMC6869321 31832232

[2] Kolahalam L.A., Kasi Viswanath I.V., Diwakar B.S., Govindh B., Reddy V. and Murthy Y.L.N. (2019) Review on nanomaterials: synthesis and applications. Materials Today: Proc. 18, 2182–2190 10.1016/j.matpr.2019.07.371

[3] Mabrouk M., Das D.B., Salem Z.A. and Beherei H.H. (2021) Nanomaterials for biomedical applications: production, characterisations, recent trends and difficulties. Molecules 26, 1077 10.3390/molecules26041077 33670668 PMC7922738

[4] Li C., Wang J., Wang Y., Gao H., Wei G., Huang Y. et al. (2019) Recent progress in drug delivery. Acta Pharmaceutica Sinica B 9, 1145–1162 10.1016/j.apsb.2019.08.003 31867161 PMC6900554

[5] Hoshyar N., Gray S., Han H. and Bao G. (2016) The effect of nanoparticle size on in vivo pharmacokinetics and cellular interaction. Nanomedicine (Lond.) 11, 673–692 10.2217/nnm.16.5 27003448 PMC5561790

[6] De Jong W.H. and Borm P.J. (2008) Drug delivery and nanoparticles:applications and hazards. Int. J. Nanomed. 3, 133–149 10.2147/IJN.S596 PMC2527668 18686775

[7] Mitchell M.J., Billingsley M.M., Haley R.M., Wechsler M.E., Peppas N.A. and Langer R. (2021) Engineering precision nanoparticles for drug delivery. Nat. Rev. Drug Discov. 20, 101–124 10.1038/s41573-020-0090-8 33277608 PMC7717100

[8] Han X., Xu K., Taratula O. and Farsad K. (2019) Applications of nanoparticles in biomedical imaging. Nanoscale 11, 799–819 10.1039/C8NR07769J 30603750 PMC8112886

[9] Patra J.K., Das G., Fraceto L.F., Campos E.V.R., Rodriguez-Torres M.D.P., Acosta-Torres L.S. et al. (2018) Nano based drug delivery systems: recent developments and future prospects. J. Nanobiotechnol. 16, 71 10.1186/s12951-018-0392-8 PMC6145203 30231877

[10] Fu X., Shi Y., Qi T., Qiu S., Huang Y., Zhao X. et al. (2020) Precise design strategies of nanomedicine for improving cancer therapeutic efficacy using subcellular targeting. Signal Transduct. Target Ther. 5, 262 10.1038/s41392-020-00342-0 33154350 PMC7644763

[11] Kim D., Jeong Y.Y. and Jon S. (2010) A drug-loaded aptamer-gold nanoparticle bioconjugate for combined CT imaging and therapy of prostate cancer. ACS Nano 4, 3689–3696 10.1021/nn901877h 20550178

[12] Giljohann D.A., Seferos D.S., Daniel W.L., Massich M.D., Patel P.C. and Mirkin C.A. (2010) Gold nanoparticles for biology and medicine. Angew. Chem. Int. Ed. Engl. 49, 3280–3294 10.1002/anie.200904359 20401880 PMC3930332

[13] Roth J. (1996) Protein glycosylation in the endoplasmic reticulum and the Golgi apparatus and cell type-specificity of cell surface glycoconjugate expression: analysis by the protein A-gold and lectin-gold techniques. Histochem. Cell Biol. 106, 79–92 10.1007/BF02473203 8858368

[14] Sperling R.A., Rivera Gil P., Zhang F., Zanella M. and Parak W.J. (2008) Biological applications of gold nanoparticles. Chem. Soc. Rev. 37, 1896–1908 10.1039/b712170a 18762838

[15] He H., Xie C. and Ren J. (2008) Nonbleaching fluorescence of gold nanoparticles and its applications in cancer cell imaging. Anal. Chem. 80, 5951–5957 10.1021/ac8005796 18590338

[16] Larson T.A., Bankson J., Aaron J. and Sokolov K. (2007) Hybrid plasmonic magnetic nanoparticles as molecular specific agents for MRI/optical imaging and photothermal therapy of cancer cells. Nanotechnology 18, 325101 10.1088/0957-4484/18/32/325101

[17] Silvestri A., Di Silvio D., Llarena I., Murray R.A., Marelli M., Lay L. et al. (2017) Influence of surface coating on the intracellular behaviour of gold nanoparticles: a fluorescence correlation spectroscopy study. Nanoscale 9, 14730–14739 10.1039/C7NR04640E 28948261

[18] Debouttière P.-J., Roux S., Vocanson F., Billotey C., Beuf O., Favre-Réguillon A. et al. (2006) Design of gold nanoparticles for magnetic resonance imaging. Adv. Funct. Mater. 16, 2330–2339 10.1002/adfm.200600242

[19] Wang Z., Qiao R., Tang N., Lu Z., Wang H., Zhang Z. et al. (2017) Active targeting theranostic iron oxide nanoparticles for MRI and magnetic resonance-guided focused ultrasound ablation of lung cancer. Biomaterials 127, 25–35 10.1016/j.biomaterials.2017.02.037 28279919 PMC5400286

[20] Singh A.P., Biswas A., Shukla A. and Maiti P. (2019) Targeted therapy in chronic diseases using nanomaterial-based drug delivery vehicles. Signal Transduct Target Ther. 4, 33 10.1038/s41392-019-0068-3 31637012 PMC6799838

[21] Rahman M.M., Islam M.R., Akash S., Harun-Or-Rashid M., Ray T.K., Rahaman M.S. et al. (2022) Recent advancements of nanoparticles application in cancer and neurodegenerative disorders: At a glance. Biomed. Pharmacother. 153, 113305 10.1016/j.biopha.2022.113305 35717779

[22] Wang L., Hu C. and Shao L. (2017) The antimicrobial activity of nanoparticles: present situation and prospects for the future. Int. J. Nanomed. 12, 1227–1249 10.2147/IJN.S121956 PMC5317269 28243086

[23] Sharmin S., Rahaman M.M., Sarkar C., Atolani O., Islam M.T. and Adeyemi O.S. (2021) Nanoparticles as antimicrobial and antiviral agents: A literature-based perspective study. Heliyon 7, e06456 10.1016/j.heliyon.2021.e06456 33763612 PMC7973307

[24] Chowdhury A., Kunjiappan S., Panneerselvam T., Somasundaram B. and Bhattacharjee C. (2017) Nanotechnology and nanocarrier-based approaches on treatment of degenerative diseases. Int. Nano Lett. 7, 91–122 10.1007/s40089-017-0208-0

[25] Ngowi E.E., Wang Y.Z., Qian L., Helmy Y., Anyomi B., Li T. et al. (2021) The application of nanotechnology for the diagnosis and treatment of brain diseases and disorders. Front Bioeng. Biotechnol. 9, 629832 10.3389/fbioe.2021.629832 33738278 PMC7960921

[26] Singh B.N., Prateeksha, Gupta V.K., Chen J. and Atanasov A.G. (2017) Organic nanoparticle-based combinatory approaches for gene therapy. Trends Biotechnol. 35, 1121–1124 10.1016/j.tibtech.2017.07.010 28818304

[27] Bovier P.A. (2008) Epaxal: a virosomal vaccine to prevent hepatitis A infection. Expert Rev. Vaccines 7, 1141–1150 10.1586/14760584.7.8.1141 18844588

[28] Herzog C., Hartmann K., Kunzi V., Kursteiner O., Mischler R., Lazar H. et al. (2009) Eleven years of Inflexal V-a virosomal adjuvanted influenza vaccine. Vaccine 27, 4381–4387 10.1016/j.vaccine.2009.05.029 19450630

[29] Schmidt-Erfurth U. and Hasan T. (2000) Mechanisms of action of photodynamic therapy with verteporfin for the treatment of age-related macular degeneration. Surv. Ophthalmol. 45, 195–214 10.1016/S0039-6257(00)00158-2 11094244

[30] Boswell G.W., Buell D. and Bekersky I. (1998) AmBisome (liposomal amphotericin B): a comparative review. J. Clin. Pharmacol. 38, 583–592 10.1002/j.1552-4604.1998.tb04464.x 9702842

[31] Anselmo A.C. and Mitragotri S. (2016) Nanoparticles in the clinic. Bioeng. Transl. Med. 1, 10–29 10.1002/btm2.10003 29313004 PMC5689513

[32] Karantzoulis S. and Galvin J.E. (2011) Distinguishing Alzheimer’s disease from other major forms of dementia. Expert Rev. Neurotherapeutics 11, 1579–1591 10.1586/ern.11.155 PMC3225285 22014137

[33] Sahni J.K., Doggui S., Ali J., Baboota S., Dao L. and Ramassamy C. (2011) Neurotherapeutic applications of nanoparticles in Alzheimer’s disease. J. Control. Release 152, 208–231 10.1016/j.jconrel.2010.11.033 21134407

[34] Ramirez-Bermudez J. (2012) Alzheimer's disease: critical notes on the history of a medical concept. Arch. Med. Res. 43, 595–599 10.1016/j.arcmed.2012.11.008 23178566

[35] Martin-Rapun R., De Matteis L., Ambrosone A., Garcia-Embid S., Gutierrez L. and de la Fuente J.M. (2017) Targeted nanoparticles for the treatment of Alzheimer’s disease. Curr. Pharm. Des. 23, 1927–1952 10.2174/1381612822666161226151011 28025949

[36] Murphy M.P. and LeVine H. III (2010) Alzheimer's disease and the amyloid-β peptide. J. Alzheimers Dis. 19, 311–323 10.3233/JAD-2010-1221 20061647 PMC2813509

[37] Chen G.-f., Xu T.-h., Yan Y., Zhou Y.-r., Jiang Y., Melcher K. and Xu H.E. (2017) Amyloid beta: structure, biology and structure-based therapeutic development. Acta Pharmacol. Sin. 38, 1205–1235 10.1038/aps.2017.28 28713158 PMC5589967

[38] O'brien R.J. and Wong P.C. (2011) Amyloid precursor protein processing and Alzheimer's disease. Annu. Rev. Neurosci. 34, 185 10.1146/annurev-neuro-061010-113613 21456963 PMC3174086

[39] Selkoe D.J. (2001) Alzheimer's disease: genes, proteins, and therapy. Physiol. Rev. 81, 741–766 10.1152/physrev.2001.81.2.741 11274343

[40] Walsh D.M. and Selkoe D.J. (2004) Deciphering the molecular basis of memory failure in Alzheimer's disease. Neuron 44, 181–193 10.1016/j.neuron.2004.09.010 15450169

[41] Kniesel U. and Wolburg H. (2000) Tight junctions of the blood-brain barrier. Cell. Mol. Neurobiol. 20, 57–76 10.1023/A:1006995910836 10690502 PMC11537529

[42] Roney C., Kulkarni P., Arora V., Antich P., Bonte F., Wu A. et al. (2005) Targeted nanoparticles for drug delivery through the blood-brain barrier for Alzheimer's disease. J. Control. Release 108, 193–214 10.1016/j.jconrel.2005.07.024 16246446

[43] Giorgetti S., Greco C., Tortora P. and Aprile F.A. (2018) Targeting amyloid aggregation: an overview of strategies and mechanisms. Int. J. Mol. Sci. 19, 2677 10.3390/ijms19092677 30205618 PMC6164555

[44] Sauvage F., Schymkowitz J., Rousseau F., Schmidt B.Z., Remaut K., Braeckmans K. et al. (2020) Nanomaterials to avoid and destroy protein aggregates. Nano Today 31, 100837 10.1016/j.nantod.2019.100837

[45] Ramesh N.K., Sudhakar S. and Mani E. (2018) Modeling of the inhibitory effect of nanoparticles on amyloid β fibrillation. Langmuir 34, 4004–4012 10.1021/acs.langmuir.8b00388 29553751

[46] Yang L., Sun J., Xie W., Liu Y. and Liu J. (2017) Dual-functional selenium nanoparticles bind to and inhibit amyloid β fiber formation in Alzheimer's disease. J. Mater. Chem. B. 5, 5954–5967 10.1039/C6TB02952C 32264352

[47] Cabaleiro-Lago C., Quinlan-Pluck F., Lynch I., Lindman S., Minogue A.M., Thulin E. et al. (2008) Inhibition of amyloid β protein fibrillation by polymeric nanoparticles. J. Am. Chem. Soc. 130, 15437–15443 10.1021/ja8041806 18954050

[48] Liu H., Xie B., Dong X., Zhang L., Wang Y., Liu F. et al. (2016) Negatively charged hydrophobic nanoparticles inhibit amyloid β-protein fibrillation: the presence of an optimal charge density. React. Funct. Polym. 103, 108–116 10.1016/j.reactfunctpolym.2016.04.003

[49] Waku T., Kobayashi Y., Wada M., Hamawaki T., Handa A., Okuda M. et al. (2020) Inhibition of amyloid β fibrillation by nanoparticles composed of ovalbumin-derived amphiphilic peptides. Chem. Lett. 49, 383–385 10.1246/cl.200048

[50] Waris A., Ali A., Khan A.U., Asim M., Zamel D., Fatima K. et al. (2022) Applications of various types of nanomaterials for the treatment of neurological disorders. Nanomaterials 12, 2140 10.3390/nano12132140 35807977 PMC9268720

[51] Zhao J., Xu N., Yang X., Ling G. and Zhang P. (2022) The roles of gold nanoparticles in the detection of amyloid-β peptide for Alzheimer's disease. Colloid Interface Sci. Commun. 46, 100579 10.1016/j.colcom.2021.100579

[52] Wang W., Han Y., Fan Y. and Wang Y. (2019) Effects of gold nanospheres and nanocubes on amyloid-beta peptide fibrillation. Langmuir 35, 2334–2342 10.1021/acs.langmuir.8b04006 30636427

[53] Mondal S., Roy Chowdhury S., Shah M., Kumar V., Kumar S. and Iyer P.K. (2019) Nanoparticle assisted regulation of nucleation pathway of amyloid tetramer and inhibition of their fibrillation kinetics. ACS Appl. Bio. Mater. 2, 2137–2142 10.1021/acsabm.9b00128 35030652

[54] Huang Y., Chang Y., Liu L. and Wang J. (2021) Nanomaterials for modulating the aggregation of β-amyloid peptides. Molecules 26, 4301 10.3390/molecules26144301 34299575 PMC8305396

[55] Bastús N.G., Comenge J. and Puntes V. (2011) Kinetically controlled seeded growth synthesis of citrate-stabilized gold nanoparticles of up to 200 nm: size focusing versus Ostwald ripening. Langmuir 27, 11098–11105 10.1021/la201938u 21728302

[56] Moore K.A., Pate K.M., Soto-Ortega D.D., Lohse S., van der Munnik N., Lim M. et al. (2017) Influence of gold nanoparticle surface chemistry and diameter upon Alzheimer's disease amyloid-β protein aggregation. J. Biological Eng. 11, 1–11 10.1186/s13036-017-0047-6 PMC5292815 28191036

[57] Shi P., Li M., Ren J. and Qu X. (2013) Gold nanocage‐based dual responsive “caged metal chelator” release system: noninvasive remote control with near infrared for potential treatment of Alzheimer's disease. Adv. Funct. Mater. 23, 5412–5419 10.1002/adfm.201301015

[58] Yin T., Xie W., Sun J., Yang L. and Liu J. (2016) Penetratin peptide-functionalized gold nanostars: enhanced BBB permeability and NIR photothermal treatment of Alzheimer's disease using ultralow irradiance. ACS Appl. Mater. Interfaces 8, 19291–19302 10.1021/acsami.6b05089 27411476

[59] Xiong N., Zhao Y., Dong X., Zheng J. and Sun Y. (2017) Design of a molecular hybrid of dual peptide inhibitors coupled on AuNPs for enhanced inhibition of amyloid β‐protein aggregation and cytotoxicity. Small 13, 1601666 10.1002/smll.201601666 28112856

[60] Sudhakar S. and Mani E. (2019) Rapid dissolution of amyloid β fibrils by silver nanoplates. Langmuir 35, 6962–6970 10.1021/acs.langmuir.9b00080 31030521

[61] Qi Y., Yi P., He T., Song X., Liu Y., Li Q. et al. (2020) Quercetin-loaded selenium nanoparticles inhibit amyloid-β aggregation and exhibit antioxidant activity. Colloids Surf. A 602, 125058 10.1016/j.colsurfa.2020.125058

[62] Thakur G., Micic M., Yang Y., Li W., Movia D., Giordani S. et al. (2011) Conjugated quantum dots inhibit the amyloid β (1-42) fibrillation process. Int. J. Alzheimer’s Dis. 2011, 1–15 10.4061/2011/502386 PMC3056432 21423556

[63] Halevas E., Mavroidi B., Nday C.M., Tang J., Smith G.C., Boukos N. et al. (2020) Modified magnetic core-shell mesoporous silica nano-formulations with encapsulated quercetin exhibit anti-amyloid and antioxidant activity. J. Inorg. Biochem. 213, 111271 10.1016/j.jinorgbio.2020.111271 33069945

[64] Dilnawaz F. and Sahoo S.K. (2015) Therapeutic approaches of magnetic nanoparticles for the central nervous system. Drug Discov. Today 20, 1256–1264 10.1016/j.drudis.2015.06.008 26103617

[65] Vrignaud S., Benoit J.-P. and Saulnier P. (2011) Strategies for the nanoencapsulation of hydrophilic molecules in polymer-based nanoparticles. Biomaterials 32, 8593–8604 10.1016/j.biomaterials.2011.07.057 21831421

[66] Geng H., Pan Y.c., Zhang R., Gao D., Wang Z., Li B. et al. (2021) Binding to Amyloid‐β Protein by Photothermal Blood‐Brain Barrier‐Penetrating Nanoparticles for Inhibition and Disaggregation of Fibrillation. Adv. Funct. Mater. 31, 2102953 10.1002/adfm.202102953

[67] Abbas M. (2021) Potential role of nanoparticles in treating the accumulation of amyloid-beta peptide in Alzheimer's patients. Polymers 13, 1051 10.3390/polym13071051 33801619 PMC8036916

[68] Ye Z., Wei L., Li Y. and Xiao L. (2019) Efficient modulation of β-amyloid peptide fibrillation with polymer nanoparticles revealed by super-resolution optical microscopy. Anal. Chem. 91, 8582–8590 10.1021/acs.analchem.9b01877 31148450

[69] Saraiva A.M., Cardoso I., Pereira M.C., Coelho M.A., Saraiva M.J., Möhwald H. et al. (2010) Controlling Amyloid‐β peptide (1-42) oligomerization and toxicity by fluorinated nanoparticles. Chem. Bio. Chem. 11, 1905–1913 10.1002/cbic.201000237 20661987

[70] Lu X., Ji C., Xu H., Li X., Ding H., Ye M. et al. (2009) Resveratrol-loaded polymeric micelles protect cells from Aβ-induced oxidative stress. Int. J. Pharm. 375, 89–96 10.1016/j.ijpharm.2009.03.021 19481694

[71] Mulik R.S., Monkkonen J., Juvonen R.O., Mahadik K.R. and Paradkar A.R. (2010) ApoE3 mediated poly (butyl) cyanoacrylate nanoparticles containing curcumin: study of enhanced activity of curcumin against beta amyloid induced cytotoxicity using in vitro cell culture model. Mol. Pharm. 7, 815–825 10.1021/mp900306x 20230014

[72] Agyare E.K., Curran G.L., Ramakrishnan M., Yu C.C., Poduslo J.F. and Kandimalla K.K. (2008) Development of a smart nano-vehicle to target cerebrovascular amyloid deposits and brain parenchymal plaques observed in Alzheimer's disease and cerebral amyloid angiopathy. Pharm. Res. 25, 2674–2684 10.1007/s11095-008-9688-y 18712585 PMC3766361

[73] Sengul A.B. and Asmatulu E. (2020) Toxicity of metal and metal oxide nanoparticles: a review. Environ. Chem. Lett. 18, 1659–1683 10.1007/s10311-020-01033-6

[74] Taylor M., Moore S., Mourtas S., Niarakis A., Re F., Zona C. et al. (2011) Effect of curcumin-associated and lipid ligand-functionalized nanoliposomes on aggregation of the Alzheimer's Aβ peptide. Nanomed. Nanotechnol. Biol. Med. 7, 541–550 10.1016/j.nano.2011.06.015 21722618

[75] Li M., Xu C., Wu L., Ren J., Wang E. and Qu X. (2013) Self‐Assembled Peptide-Polyoxometalate Hybrid Nanospheres: Two in One Enhances Targeted Inhibition of Amyloid β‐Peptide Aggregation Associated with Alzheimer's Disease. Small 9, 3455–3461 10.1002/smll.201202612 23650245

[76] Liu L., Chang Y., Yu J., Jiang M. and Xia N. (2017) Two-in-one polydopamine nanospheres for fluorescent determination of beta-amyloid oligomers and inhibition of beta-amyloid aggregation. Sens. Actuators B 251, 359–365 10.1016/j.snb.2017.05.106

[77] Panda A.K., Chakraborty A., Nandi S.K., Kaushik A. and Biswas A. (2017) The C‐terminal extension of Mycobacterium tuberculosis Hsp16. 3 regulates its oligomerization, subunit exchange dynamics and chaperone function. FEBS J. 284, 277–300 10.1111/febs.13975 27885799

[78] Organization WH. (2020) Global tuberculosis report 2021, World Health Organization, Geneva, 2021. Licence: CC BY-NC-SA 3.0 IGO

[79] Nasiruddin M., Neyaz M. and Das S. (2017) Nanotechnology-based approach in tuberculosis treatment. Tuberculosis Res. Treatment 2017, 2231–2258 10.1155/2017/4920209 PMC5292193 28210505

[80] Matsuo K. and Yasutomi Y. (2011) Mycobacterium bovis Bacille Calmette-Guerin as a vaccine vector for global infectious disease control. Tuberculosis Res. Treatment 2011, 1–9 10.1155/2011/574591 PMC3335490 22567267

[81] Chen C.-C., Chen Y.-Y., Yeh C.-C., Hsu C.-W., Yu S.-J., Hsu C.-H. et al. (2021) Alginate-capped silver nanoparticles as a potent anti-mycobacterial agent against mycobacterium tuberculosis. Front. Pharmacol. 12, 1–15 10.3389/fphar.2021.746496 PMC8660078 34899300

[82] Tăbăran A.-F., Matea C.T., Mocan T., Tăbăran A., Mihaiu M., Iancu C. et al. (2020)Silver nanoparticles for the therapy of tuberculosis. Int. J. Nanomed. 15, 2231 10.2147/IJN.S241183 PMC7127828 32280217

[83] Jafari A., Nagheli A., Foumani A.A., Soltani B. and Goswami R. (2020) The role of metallic nanoparticles in inhibition of Mycobacterium tuberculosis and enhances phagosome maturation into the infected macrophage. Oman Med. J. 35, e194 10.5001/omj.2020.78 33214909 PMC7658918

[84] Morales-Avila E., Ferro-Flores G., Ocampo-García B.E., López-Téllez G., López-Ortega J., Rogel-Ayala D.G. et al. (2017) Antibacterial efficacy of gold and silver nanoparticles functionalized with the ubiquicidin (29-41) antimicrobial peptide. J. Nanomaterials 2017, 1–10 10.1155/2017/5831959

[85] Kalmantaeva O., Firstova V., Grishchenko N., Rudnitskaya T., Potapov V. and Ignatov S. (2020) Antibacterial and immunomodulating activity of silver nanoparticles on mice experimental tuberculosis model. Appl. Biochem. Microbiol. 56, 226–232 10.1134/S0003683820020088

[86] Zhou Y., Kong Y., Kundu S., Cirillo J.D. and Liang H. (2012) Antibacterial activities of gold and silver nanoparticles against Escherichia coli and bacillus Calmette-Guérin. J. Nanobiotechnol. 10, 1–9 10.1186/1477-3155-10-19 PMC3405418 22559747

[87] Montalvo-Quirós S., Gómez-Graña S., Vallet-Regí M., Prados-Rosales R.C., González B. and Luque-Garcia J.L. (2021) Mesoporous silica nanoparticles containing silver as novel antimycobacterial agents against Mycobacterium tuberculosis. Colloids Surf. B 197, 111405 10.1016/j.colsurfb.2020.111405 33130523

[88] Singh R., Nawale L., Arkile M., Wadhwani S., Shedbalkar U., Chopade S. et al. (2016) Phytogenic silver, gold, and bimetallic nanoparticles as novel antitubercular agents. Int. J. Nanomed. 11, 1889 10.2147/IJN.S102488 PMC4862349 27217751

[89] Ellis T., Chiappi M., Garcia-Trenco A., Al-Ejji M., Sarkar S., Georgiou T.K. et al. (2018) Multimetallic Microparticles Increase the Potency of Rifampicin against Intracellular Mycobacterium tuberculosis. ACS Nano 12, 5228–5240 10.1021/acsnano.7b08264 29767993

[90] Ali H.R., Ali M.R., Wu Y., Selim S.A., Abdelaal H.F., Nasr E.A. et al. (2016) Gold nanorods as drug delivery vehicles for rifampicin greatly improve the efficacy of combating Mycobacterium tuberculosis with good biocompatibility with the host cells. Bioconjug. Chem. 27, 2486–2492 10.1021/acs.bioconjchem.6b00430 27595304

[91] Sun F., Oh S., Kim J., Kato T., Kim H.-J., Lee J. et al. (2017) Enhanced internalization of macromolecular drugs into mycobacterium smegmatis with the assistance of silver nanoparticles. J. Microbiol. Biotechnol. 27, 1483–1490 10.4014/jmb.1612.12041 28595381

[92] Kreytsberg G., Gracheva I., Kibrik B. and Golikov I. (2011) Antituberculous effect of silver nanoparticles. J. Physics: Conference Series, IOP Publishing 291, 012030, pp 10.1088/1742-6596/291/1/012030

[93] Chakraborty A., Ghosh R. and Biswas A. (2022) Interaction of constituents of MDT regimen for leprosy with Mycobacterium leprae HSP18: impact on its structure and function. FEBS J. 289, 832–853 10.1111/febs.16212 34555271

[94] Schneider-Rauber G., Argenta D.F. and Caon T. (2020) Emerging Technologies to Target Drug Delivery to the Skin - the Role of Crystals and Carrier-Based Systems in the Case Study of Dapsone. Pharm. Res. 37, 240 10.1007/s11095-020-02951-4 33169237

[95] Joshi G., Quadir S.S. and Yadav K.S. (2021) Road map to the treatment of neglected tropical diseases: nanocarriers interventions. J. Control. Release 339, 51–74 10.1016/j.jconrel.2021.09.020 34555491

[96] Chaves L.L., Costa Lima S.A., Vieira A.C., Barreiros L., Segundo M.A., Ferreira D. et al. (2017) pH-sensitive nanoparticles for improved oral delivery of dapsone: risk assessment, design, optimization and characterization. Nanomedicine (Lond) 12, 1975–1990 10.2217/nnm-2017-0105 28745104

[97] Chaves L.L., Costa Lima S.A., Vieira A.C.C., Barreiros L., Segundo M.A., Ferreira D. et al. (2018) Development of PLGA nanoparticles loaded with clofazimine for oral delivery: Assessment of formulation variables and intestinal permeability. Eur. J. Pharm. Sci. 112, 28–37 10.1016/j.ejps.2017.11.004 29122712

[98] Chaves L.L., Costa Lima S.A., Vieira A.C.C., Barreiros L., Segundo M.A., Ferreira D. et al. (2018) Nanosystems as modulators of intestinal dapsone and clofazimine delivery. Biomed. Pharmacother. 103, 1392–1396 10.1016/j.biopha.2018.04.195 29864923

[99] Chen W., Cheng C.A., Lee B.Y., Clemens D.L., Huang W.Y., Horwitz M.A. et al. (2018) Facile strategy enabling both high loading and high release amounts of the water-insoluble drug clofazimine using mesoporous silica nanoparticles. ACS Appl. Mater. Interfaces 10, 31870–31881 10.1021/acsami.8b09069 30160469

[100] Vieira A.C., Chaves L.L., Pinheiro M., Ferreira D., Sarmento B. and Reis S. (2016) Design and statistical modeling of mannose-decorated dapsone-containing nanoparticles as a strategy of targeting intestinal M-cells. Int. J. Nanomed. 11, 2601–2617 10.2147/IJN.S104908 PMC4907709 27354792

[101] Kanwar R., Gradzielski M. and Mehta S.K. (2018) Biomimetic solid lipid nanoparticles of sophorolipids designed for antileprosy drugs. J. Phys. Chem. B 122, 6837–6845 10.1021/acs.jpcb.8b03081 29874078

[102] de Melo Barbosa R., Meirelles L.M.A., García-Villén F., Câmara G.B.M., Finkler C.L.L., Iborra C.V. et al. (2021) Lipid nanoparticles for the treatment of neglected tropical diseases. Applications of Nanobiotechnology for Neglected Tropical Diseases, pp. 357–377, Elsevier

[103] Monteiro L.M., Lione V.F., do Carmo F.A., do Amaral L.H., da Silva J.H., Nasciutti L.E. et al. (2012) Development and characterization of a new oral dapsone nanoemulsion system: permeability and in silico bioavailability studies. Int. J. Nanomed. 7, 5175–5182 10.2147/IJN.S36479 PMC3463397 23055729

[104] Islan G.A., Duran M., Cacicedo M.L., Nakazato G., Kobayashi R.K.T., Martinez D.S.T. et al. (2017) Nanopharmaceuticals as a solution to neglected diseases: Is it possible? Acta Trop. 170, 16–42 10.1016/j.actatropica.2017.02.019 28232069

[105] Borges V.R., Simon A., Sena A.R., Cabral L.M. and de Sousa V.P. (2013) Nanoemulsion containing dapsone for topical administration: a study of in vitro release and epidermal permeation. Int. J. Nanomed. 8, 535–544 10.2147/IJN.S39383 PMC3572825 23411489

[106] Taufikurohmah T., Soepardjo D., Armadianto H. and Rusmini R. (2019) Synthesis and Characterization of Nanogold and Nanosilver as Leprosy Drug Candidates and Their Activity Tests in Leprosy Patients; Case Study. Mathematics, Informatics, Science, and Education International Conference (MISEIC 2019), pp. 221–226, Atlantis Press 10.2991/miseic-19.2019.6

[107] Chakraborty A., Nandi S.K., Panda A.K., Mahapatra P.P., Giri S. and Biswas A. (2018) Probing the structure-function relationship of Mycobacterium leprae HSP18 under different UV radiations. Int. J. Biol. Macromol. 119, 604–616 10.1016/j.ijbiomac.2018.07.151 30055280

[108] Chakraborty A. and Biswas A. (2020) Structure, stability and chaperone function of Mycobacterium leprae Heat Shock Protein 18 are differentially affected upon interaction with gold and silver nanoparticles. Int. J. Biol. Macromol. 152, 250–260 10.1016/j.ijbiomac.2020.02.182 32084461

[109] Meacham C.E. and Morrison S.J. (2013) Tumour heterogeneity and cancer cell plasticity. Nature 501, 328–337 10.1038/nature12624 24048065 PMC4521623

[110] Fisher R., Pusztai L. and Swanton C. (2013) Cancer heterogeneity: implications for targeted therapeutics. Br. J. Cancer 108, 479–485 10.1038/bjc.2012.581 23299535 PMC3593543

[111] Mansoori B., Mohammadi A., Davudian S., Shirjang S. and Baradaran B. (2017) The different mechanisms of cancer drug resistance: a brief review. Adv. Pharm. Bull 7, 339–348 10.15171/apb.2017.041 29071215 PMC5651054

[112] Cheng Y., He C., Wang M., Ma X., Mo F., Yang S. et al. (2019) Targeting epigenetic regulators for cancer therapy: mechanisms and advances in clinical trials. Signal Transduct. Target Ther. 4, 62 10.1038/s41392-019-0095-0 31871779 PMC6915746

[113] Hoter A., El-Sabban M.E. and Naim H.Y. (2018) The HSP90 family: structure, regulation, function, and implications in health and disease. Int. J. Mol. Sci. 19, 2560 10.3390/ijms19092560 30158430 PMC6164434

[114] Zuo D., Subjeck J. and Wang X.Y. (2016) Unfolding the role of large heat shock proteins: new insights and therapeutic implications. Front. Immunol. 7, 75 10.3389/fimmu.2016.00075 26973652 PMC4771732

[115] Nagaraju G.P., Long T.E., Park W., Landry J.C., Taliaferro-Smith L., Farris A.B. et al. (2015) Heat shock protein 90 promotes epithelial to mesenchymal transition, invasion, and migration in colorectal cancer. Mol. Carcinog. 54, 1147–1158 10.1002/mc.22185 24861206

[116] Chatterjee S., Bhattacharya S., Socinski M.A. and Burns T.F. (2016) HSP90 inhibitors in lung cancer: promise still unfulfilled. Clin. Adv. Hematol. Oncol. 14, 346–356 27379696

[117] Wu J., Liu T., Rios Z., Mei Q., Lin X. and Cao S. (2017) Heat shock proteins and cancer. Trends Pharmacol. Sci. 38, 226–256 10.1016/j.tips.2016.11.009 28012700

[118] Meng J., Liu Y., Han J., Tan Q., Chen S., Qiao K. et al. (2017) Hsp90beta promoted endothelial cell-dependent tumor angiogenesis in hepatocellular carcinoma. Mol. Cancer 16, 72 10.1186/s12943-017-0640-9 28359326 PMC5374580

[119] McCarthy M.M., Pick E., Kluger Y., Gould-Rothberg B., Lazova R., Camp R.L. et al. (2008) HSP90 as a marker of progression in melanoma. Ann. Oncol. 19, 590–594 10.1093/annonc/mdm545 18037622

[120] Zackova M., Mouckova D., Lopotova T., Ondrackova Z., Klamova H. and Moravcova J. (2013) Hsp90 - a potential prognostic marker in CML. Blood Cells Mol. Dis. 50, 184–189 10.1016/j.bcmd.2012.11.002 23190580

[121] Liu K., Kang M., Li J., Qin W. and Wang R. (2019) Prognostic value of the mRNA expression of members of the HSP90 family in non-small cell lung cancer. Exp. Ther. Med. 17, 2657–2665 10.3892/etm.2019.7228 30930968 PMC6425268

[122] Lin T., Qiu Y., Peng W. and Peng L. (2020) Heat shock protein 90 family isoforms as prognostic biomarkers and their correlations with immune infiltration in breast cancer. Biomed. Res. Int. 2020, 2148253 10.1155/2020/2148253 33145341 PMC7596464

[123] Kamal A., Thao L., Sensintaffar J., Zhang L., Boehm M.F., Fritz L.C. et al. (2003) A high-affinity conformation of Hsp90 confers tumour selectivity on Hsp90 inhibitors. Nature 425, 407–410 10.1038/nature01913 14508491

[124] Roe S.M., Prodromou C., O'Brien R., Ladbury J.E., Piper P.W. and Pearl L.H. (1999) Structural basis for inhibition of the Hsp90 molecular chaperone by the antitumor antibiotics radicicol and geldanamycin. J. Med. Chem. 42, 260–266 10.1021/jm980403y 9925731

[125] Schulte T.W., Akinaga S., Soga S., Sullivan W., Stensgard B., Toft D. et al. (1998) Antibiotic radicicol binds to the N-terminal domain of Hsp90 and shares important biologic activities with geldanamycin. Cell Stress Chaperones 3, 100–108 10.1379/1466-1268(1998)003<0100:ARBTTN>2.3.CO;2 9672245 PMC312953

[126] Rastelli G., Tian Z.Q., Wang Z., Myles D. and Liu Y. (2005) Structure-based design of 7-carbamate analogs of geldanamycin. Bioorg. Med. Chem. Lett. 15, 5016–5021 10.1016/j.bmcl.2005.08.013 16165354

[127] Marcu M.G., Chadli A., Bouhouche I., Catelli M. and Neckers L.M. (2000) The heat shock protein 90 antagonist novobiocin interacts with a previously unrecognized ATP-binding domain in the carboxyl terminus of the chaperone. J. Biol. Chem. 275, 37181–37186 10.1074/jbc.M003701200 10945979

[128] Audisio D., Messaoudi S., Cegielkowski L., Peyrat J.F., Brion J.D., Methy-Gonnot D. et al. (2011) Discovery and biological activity of 6BrCaQ as an inhibitor of the Hsp90 protein folding machinery. Chem. Med. Chem. 6, 804–815 10.1002/cmdc.201000489 21374821

[129] Xiong M.P., Yanez J.A., Kwon G.S., Davies N.M. and Forrest M.L. (2009) A cremophor-free formulation for tanespimycin (17-AAG) using PEO-b-PDLLA micelles: characterization and pharmacokinetics in rats. J. Pharm. Sci. 98, 1577–1586 10.1002/jps.21509 18752263 PMC2649998

[130] Chandran T., Katragadda U., Teng Q. and Tan C. (2010) Design and evaluation of micellar nanocarriers for 17-allyamino-17-demethoxygeldanamycin (17-AAG). Int. J. Pharm. 392, 170–177 10.1016/j.ijpharm.2010.03.056 20363305

[131] Saxena V., Naguib Y. and Hussain M.D. (2012) Folate receptor targeted 17-allylamino-17-demethoxygeldanamycin (17-AAG) loaded polymeric nanoparticles for breast cancer. Colloids Surf. B Biointerfaces 94, 274–280 10.1016/j.colsurfb.2012.02.001 22377218

[132] Saxena V. and Hussain M.D. (2013) Formulation and in vitro evaluation of 17-allyamino-17-demethoxygeldanamycin (17-AAG) loaded polymeric mixed micelles for glioblastoma multiforme. Colloids Surf. B Biointerfaces 112, 350–355 10.1016/j.colsurfb.2013.07.031 24012704

[133] Larson N., Greish K., Bauer H., Maeda H. and Ghandehari H. (2011) Synthesis and evaluation of poly(styrene-co-maleic acid) micellar nanocarriers for the delivery of tanespimycin. Int. J. Pharm. 420, 111–117 10.1016/j.ijpharm.2011.08.011 21856392 PMC3195848

[134] Saif M.W., Erlichman C., Dragovich T., Mendelson D., Toft D., Burrows F. et al. (2013) Open-label, dose-escalation, safety, pharmacokinetic, and pharmacodynamic study of intravenously administered CNF1010 (17-(allylamino)-17-demethoxygeldanamycin [17-AAG]) in patients with solid tumors. Cancer Chemother. Pharmacol. 71, 1345–1355 10.1007/s00280-013-2134-9 23564374

[135] Tao C., Yu C., De T.K., Everett N., Frankel T., Ci S. et al. (2005) Preparation of nanoparticle albumin bound 17AAG (nab-17AAG) suitable for intravenous administration. Cancer Res. 65, 336–336

[136] Won Y.W., Yoon S.M., Sonn C.H., Lee K.M. and Kim Y.H. (2011) Nano self-assembly of recombinant human gelatin conjugated with alpha-tocopheryl succinate for Hsp90 inhibitor, 17-AAG, delivery. ACS Nano 5, 3839–3848 10.1021/nn200173u 21517103

[137] Onyuksel H., Mohanty P.S. and Rubinstein I. (2009) VIP-grafted sterically stabilized phospholipid nanomicellar 17-allylamino-17-demethoxy geldanamycin: a novel targeted nanomedicine for breast cancer. Int. J. Pharm. 365, 157–161 10.1016/j.ijpharm.2008.08.024 18793708 PMC2631986

[138] Shin H.C., Alani A.W., Cho H., Bae Y., Kolesar J.M. and Kwon G.S. (2011) A 3-in-1 polymeric micelle nanocontainer for poorly water-soluble drugs. Mol. Pharm. 8, 1257–1265 10.1021/mp2000549 21630670 PMC4896148

[139] Hasenstein J.R., Shin H.C., Kasmerchak K., Buehler D., Kwon G.S. and Kozak K.R. (2012) Antitumor activity of Triolimus: a novel multidrug-loaded micelle containing Paclitaxel, Rapamycin, and 17-AAG. Mol. Cancer Ther. 11, 2233–2242 10.1158/1535-7163.MCT-11-0987 22896668 PMC3469732

[140] Katragadda U., Teng Q., Rayaprolu B.M., Chandran T. and Tan C. (2011) Multi-drug delivery to tumor cells via micellar nanocarriers. Int. J. Pharm. 419, 281–286 10.1016/j.ijpharm.2011.07.033 21820041 PMC3186853

[141] Pradhan R., Ramasamy T., Choi J.Y., Kim J.H., Poudel B.K., Tak J.W. et al. (2015) Hyaluronic acid-decorated poly(lactic-co-glycolic acid) nanoparticles for combined delivery of docetaxel and tanespimycin. Carbohydr. Polym. 123, 313–323 10.1016/j.carbpol.2015.01.064 25843864

[142] Desale S.S., Raja S.M., Kim J.O., Mohapatra B., Soni K.S., Luan H. et al. (2015) Polypeptide-based nanogels co-encapsulating a synergistic combination of doxorubicin with 17-AAG show potent anti-tumor activity in ErbB2-driven breast cancer models. J. Control. Release 208, 59–66 10.1016/j.jconrel.2015.02.001 25660204 PMC4430376

[143] Xiong M.P., Yanez J.A., Remsberg C.M., Ohgami Y., Kwon G.S., Davies N.M. et al. (2008) Formulation of a geldanamycin prodrug in mPEG-b-PCL micelles greatly enhances tolerability and pharmacokinetics in rats. J. Control. Release 129, 33–40 10.1016/j.jconrel.2008.03.015 18456363 PMC2492396

[144] Mellatyar H., Akbarzadeh A., Rahmati M., Ghalhar M.G., Etemadi A., Nejati-Koshki K. et al. (2014) Comparison of inhibitory effect of 17-DMAG nanoparticles and free 17-DMAG in HSP90 gene expression in lung cancer. Asian Pac. J. Cancer Prev. 15, 8693–8698 10.7314/APJCP.2014.15.20.8693 25374192

[145] Mellatyar H., Talaei S., Nejati-Koshki K. and Akbarzadeh A. (2016) Targeting HSP90 gene expression with 17-DMAG nanoparticles in breast cancer cells. Asian Pac. J. Cancer Prev. 17, 2453–2457 27268613

[146] Dong D., Wang X., Wang H., Zhang X., Wang Y. and Wu B. (2015) Elucidating the in vivo fate of nanocrystals using a physiologically based pharmacokinetic model: a case study with the anticancer agent SNX-2112. Int. J. Nanomed. 10, 2521–2535 10.2147/IJN.S79734 PMC4386773 25848269

[147] Zhang X., Zhang T., Ye Y., Chen H., Sun H., Zhou X. et al. (2015) Phospholipid-stabilized mesoporous carbon nanospheres as versatile carriers for systemic delivery of amphiphobic SNX-2112 (a Hsp90 inhibitor) with enhanced antitumor effect. Eur. J. Pharm. Biopharm. 94, 30–41 10.1016/j.ejpb.2015.04.023 25936860

[148] Kim S.J., Ramsey D.M., Boyer C., Davis T.P. and McAlpine S.R. (2013) Effectively delivering a unique hsp90 inhibitor using star polymers. ACS Med. Chem. Lett. 4, 10.1021/ml400082b PMC3873631 24379910

[149] Sauvage F., Franze S., Bruneau A., Alami M., Denis S., Nicolas V. et al. (2016) Formulation and in vitro efficacy of liposomes containing the Hsp90 inhibitor 6BrCaQ in prostate cancer cells. Int. J. Pharm. 499, 101–109 10.1016/j.ijpharm.2015.12.053 26721724

[150] Pore S.K., Choudhary A., Rathore B., Ganguly A., Sujitha P., Kumar C.G. et al. (2013) Hsp90-targeted miRNA-liposomal formulation for systemic antitumor effect. Biomaterials 34, 6804–6817 10.1016/j.biomaterials.2013.05.054 23773821

[151] Yavelsky V., Vais O., Piura B., Wolfson M., Rabinovich A. and Fraifeld V. (2004) The role of Hsp90 in cell response to hyperthermia. J. Therm. Biol 29, 509–514 10.1016/j.jtherbio.2004.08.078

[152] Bae Y., Buresh R.A., Williamson T.P., Chen T.H. and Furgeson D.Y. (2007) Intelligent biosynthetic nanobiomaterials for hyperthermic combination chemotherapy and thermal drug targeting of HSP90 inhibitor geldanamycin. J. Control. Release 122, 16–23 10.1016/j.jconrel.2007.06.005 17651857

[153] Song Y., Wang Y., Zhu Y., Cheng Y., Wang Y., Wang S. et al. (2019) Biomodal tumor-targeted and redox-responsive Bi2 Se3 hollow nanocubes for MSOT/CT imaging guided synergistic low-temperature photothermal radiotherapy. Adv. Healthc. Mater. 8, e1900250 10.1002/adhm.201900250 31290616

[154] Chen L., Fujisawa N., Takanohashi M., Najmina M., Uto K. and Ebara M. (2021) A smart hyperthermia nanofiber-platform-enabled sustained release of doxorubicin and 17AAG for synergistic cancer therapy. Int. J. Mol. Sci. 22, 2542 10.3390/ijms22052542 33802613 PMC7961598

[155] Zhang T., Wu B., Akakuru O.U., Yao C., Sun S., Chen L. et al. (2021) Hsp90 inhibitor-loaded IR780 micelles for mitochondria-targeted mild-temperature photothermal therapy in xenograft models of human breast cancer. Cancer Lett. 500, 41–50 10.1016/j.canlet.2020.12.028 33359275

[156] Jiang A., Liu Y., Ma L., Mao F., Liu L., Zhai X. et al. (2019) Biocompatible heat-shock protein inhibitor-delivered flowerlike short-wave infrared nanoprobe for mild temperature-driven highly efficient tumor ablation. ACS Appl. Mater. Interfaces 11, 6820–6828 10.1021/acsami.8b21483 30677285

[157] Yang R., Tang Q., Miao F., An Y., Li M., Han Y. et al. (2015) Inhibition of heat-shock protein 90 sensitizes liver cancer stem-like cells to magnetic hyperthermia and enhances anti-tumor effect on hepatocellular carcinoma-burdened nude mice. Int. J. Nanomed. 10, 7345–7358 10.2147/IJN.S93758 PMC4677660 26677324

[158] Rochani A.K., Balasubramanian S., Ravindran Girija A., Raveendran S., Borah A., Nagaoka Y. et al. (2016) Dual mode of cancer cell destruction for pancreatic cancer therapy using Hsp90 inhibitor loaded polymeric nano magnetic formulation. Int. J. Pharm. 511, 648–658 10.1016/j.ijpharm.2016.07.048 27469073

[159] Tang X., Tan L., Shi K., Peng J., Xiao Y., Li W. et al. (2018) Gold nanorods together with HSP inhibitor-VER-155008 micelles for colon cancer mild-temperature photothermal therapy. Acta. Pharm. Sin. B. 8, 587–601 10.1016/j.apsb.2018.05.011 30109183 PMC6089863

[160] You C., Li Y., Dong Y., Ning L., Zhang Y., Yao L. et al. (2020) Low-Temperature Trigger Nitric Oxide Nanogenerators for Enhanced Mild Photothermal Therapy. ACS Biomater. Sci. Eng. 6, 1535–1542 10.1021/acsbiomaterials.9b01771 33455391

[161] Bahadar H., Maqbool F., Niaz K. and Abdollahi M. (2016) Toxicity of nanoparticles and an overview of current experimental models. Iranian Biomed. J. 20, 1 10.7508/ibj.2016.01.001 PMC4689276 26286636

[162] Lee Y.K., Choi E.J., Webster T.J., Kim S.H. and Khang D. (2015) Effect of the protein corona on nanoparticles for modulating cytotoxicity and immunotoxicity. Int. J. Nanomed. 10, 97–113 10.2147/IJN.S72998 PMC4275058 25565807

[163] Kessler A., Hedberg J., Blomberg E. and Odnevall I. (2022) Reactive oxygen species formed by metal and metal oxide nanoparticles in physiological media—a review of reactions of importance to nanotoxicity and proposal for categorization. Nanomaterials 12, 1922 10.3390/nano12111922 35683777 PMC9182937

[164] Chen L.Q., Fang L., Ling J., Ding C.Z., Kang B. and Huang C.Z. (2015) Nanotoxicity of silver nanoparticles to red blood cells: size dependent adsorption, uptake, and hemolytic activity. Chem. Res. Toxicol. 28, 501–509 10.1021/tx500479m 25602487

[165] Andersson-Willman B., Gehrmann U., Cansu Z., Buerki-Thurnherr T., Krug H.F., Gabrielsson S. et al. (2012) Effects of subtoxic concentrations of TiO2 and ZnO nanoparticles on human lymphocytes, dendritic cells and exosome production. Toxicol. Appl. Pharmacol. 264, 94–103 10.1016/j.taap.2012.07.021 22842014

[166] Battal D., Çelik A., Güler G., Aktaş A., Yildirimcan S., Ocakoglu K. et al. (2015) SiO2 Nanoparticule-induced size-dependent genotoxicity - an in vitro study using sister chromatid exchange, micronucleus and comet assay. Drug Chem Toxicol. 38, 196–204 10.3109/01480545.2014.928721 24960636

[167] Magdolenova Z., Drlickova M., Henjum K., Rundén-Pran E., Tulinska J., Bilanicova D. et al. (2015) Coating-dependent induction of cytotoxicity and genotoxicity of iron oxide nanoparticles. Nanotoxicology 9, 44–56 10.3109/17435390.2013.847505 24228750

[168] Farace C., Sánchez-Moreno P., Orecchioni M., Manetti R., Sgarrella F., Asara Y. et al. (2016) Immune cell impact of three differently coated lipid nanocapsules: pluronic, chitosan and polyethylene glycol. Sci. Rep. 6, 1–14 10.1038/srep18423 26728491 PMC4700454

[169] Kim J.S., Song K.S. and Yu I.J. (2016) Multiwall carbon nanotube-induced DNA damage and cytotoxicity in male human peripheral blood lymphocytes. Int. J. Toxicol. 35, 27–37 10.1177/1091581815598749 26268766

[170] Guo C., Xia Y., Niu P., Jiang L., Duan J., Yu Y. et al. (2015) Silica nanoparticles induce oxidative stress, inflammation, and endothelial dysfunction in vitro via activation of the MAPK/Nrf2 pathway and nuclear factor-κB signaling. Int. J. Nanomed. 10, 1463 10.2147/IJN.S76114 PMC4345992 25759575

